# Dapagliflozin inhibits TGF-β-induced transdifferentiation of valvular interstitial cells and mitral valvular degeneration

**DOI:** 10.1007/s00109-025-02615-z

**Published:** 2025-12-15

**Authors:** Remi Sato, Kosei Sakai, Satoshi Kameshima

**Affiliations:** Laboratory of Small Animal Internal Medicine 1, School of Veterinary Medicine, Kitasato University, Towada City, Higashi 23 Bancho 35-1, Aomori, 034-8628 Japan

**Keywords:** Adenosine monophosphate-activated protein kinase, Dapagliflozin, Mitral valve degeneration, Smad signaling, Transforming growth factor-β, Valvular interstitial cell

## Abstract

**Abstract:**

Myxomatous mitral valve (MV) disease (MMVD) is a common cardiac condition in humans and dogs. Currently, no therapeutic agent is available for MMVD that can prevent MV degeneration. Transforming growth factor (TGF)-β-induced transdifferentiation of valvular interstitial cells (VICs) is a key process in MV degeneration. Dapagliflozin is used to treat type 2 diabetes, and it alleviates cardiac and renal fibrosis by inhibiting the TGF-β signaling pathway. In this study, we investigated the effect of dapagliflozin on TGF-β-induced transdifferentiation of VICs and MV degeneration in rats. Protein expression levels and contractile forces generated by VICs were determined using western blotting and a collagen gel contraction assay, respectively. Structural changes of the MV were detected using histological staining. Dapagliflozin inhibited TGF-β-induced Smad2/3 phosphorylation and increased alpha-smooth muscle actin expression and the cellular contractile force. In contrast, dapagliflozin increased the activity of adenosine monophosphate-activated protein kinase (AMPK)α. However, 5-aminoimidazole-4-carboxamide 1-β-D-ribofuranoside, an AMPK activator, inhibited TGF-β-induced Smad2/3 phosphorylation. AMPKα gene knockdown impeded the inhibitory effect exerted by dapagliflozin on TGF-β-induced Smad2 phosphorylation. Additionally, dapagliflozin increased AMPKα phosphorylation and suppressed TGF-β-induced MV degeneration, alpha-smooth muscle actin expression, and Smad2/3 phosphorylation in isolated rat MV. To the best of our knowledge, this study is the first to demonstrate that dapagliflozin inhibits TGF-β-induced VIC differentiation and subsequent MV degeneration by suppressing Smad signaling via AMPK activation, suggesting that dapagliflozin is a potential novel therapeutic agent for MMVD.

**Key messages:**

- TGF-β-induced VIC differentiation leads to MV degeneration.
- DAPA, an SGLT2 inhibitor, suppresses the TGF-β signaling pathway.
- DAPA inhibits TGF-β-induced differentiation of rat VIC via AMPK activation.
- DAPA inhibits TGF-β-induced degeneration and signal transduction in isolated rat MV.
- DAPA suppresses MV degeneration by inhibiting VIC differentiation.

**Supplementary Information:**

The online version contains supplementary material available at 10.1007/s00109-025-02615-z.

## Introduction

Mitral valve (MV) disease is one of the most common cardiac valvular abnormalities, affecting approximately 24.2 million people worldwide [1] and accounting for approximately 75% of heart disease cases reported for dogs in North America [2]. Myxomatous MV disease (MMVD) is caused by the myxomatous degeneration of MV, which can lead to MV prolapse. Current treatment methods for MMVD primarily focus on improving consequential systemic venous congestion using vasodilators, diuretics, and cardiotonic agents rather than on inhibiting causative MV degeneration. Therefore, new therapeutic drugs are required to prevent structural MV degeneration in MMVD.

The MV is composed of two thin flaps that include the anterior and posterior leaflets. The cross-sectional structure of the MV leaflet exhibits four distinct layers: atrialis, spongiosa, fibrosa, and ventricularis. Each layer contains varying amounts of extracellular matrix components, including collagen-I, -III, -IV, and -VI, as well as laminin, fibronectin, and heparan sulfate [3]. Both sides of the valve are lined with valvular endothelial cells [4]. Quiescent valvular interstitial cells (VICs) are the primary cellular components of normal MV [5]. Myxomatous degeneration causes MV thickening, resulting in prolapsed leaflets and MV regurgitation. Human MV prolapse is caused by rheumatic fever, infectious endocarditis, and Barlow’s disease [6, 7]. Myxomatous MV degeneration mostly occurs idiopathically in dogs, and the underlying pathogenetic mechanisms remain unknown.

Changes in the expression of molecules related to inflammation, extracellular matrix formation, and transdifferentiation have been detected using transcriptomic profiling and expression analysis in diseased MV tissues [8]. Transforming growth factor (TGF)-β, a cytokine member of the TGF-β superfamily, plays an important role in the pathogenesis of various diseases, including MMVD. TGF-β increases alpha-smooth muscle actin (α-SMA) expression by activating the Smad-dependent signaling pathways, leading to the transformation of quiescent VICs into α-SMA-positive myofibroblast-like active cells [9]. VIC transdifferentiation causes MV degeneration and valvular thickening via destruction of the layer structure and extracellular matrix remodeling involving abnormal accumulation of acid mucopolysaccharides [3]. TGF-β expression and α-SMA- and phosphorylated (p)-Smad2/3-positive cell numbers are higher in degenerative MV tissues of human patients with MMVD [10].

Dapagliflozin (DAPA), a sodium–glucose cotransporter (SGLT) 2 inhibitor, is used to treat human patients with type 2 diabetes, chronic heart failure, and kidney disease. It inhibits TGF-β-induced endothelial–mesenchymal transition by activating adenosine monophosphate-activated protein kinase (AMPK)α in human umbilical vein endothelial cells under hyperglycemic conditions [11]. Moreover, DAPA alleviates cardiac fibrosis in normoglycemic rabbits with congestive heart failure by inhibiting the TGF-β1/Smad signaling pathway [12]. In this study, we investigated its effect on TGF-β-induced transdifferentiation of VICs and MV degeneration in rats.

## Materials and methods

### Materials

The reagents used in this study included DAPA (ChemScene, Monmouth Junction, NJ, USA), recombinant human TGF-β1 (PeproTech, Rocky Hill, NJ, USA), and 5-aminoimidazole-4-carboxamide 1-β-d-ribofuranoside (AICAR; AdipoGen, San Diego, CA, USA). DAPA and AICAR were dissolved in dimethyl sulfoxide (Nacalai Tesque, Kyoto, Japan), and TGF-β1 was dissolved in 0.1% bovine serum albumin.

The antibodies used in this study included anti-α-SMA (1:1000; Agilent Technologies, Santa Clara, CA, USA), anti-p-Smad2 (1:500; Cell Signaling Technology, Danvers, MA, USA), anti-p-Smad3 (1:250; Cell Signaling Technology), anti-total (t)-Smad2/3 (1:1000 or 1:500; Cell Signaling Technology), anti-p-AMPKα (1:500; Cell Signaling Technology), anti-t-AMPKα (1:500; Cell Signaling Technology), anti-p-acetyl-CoA carboxylase (ACC; 1:500; Cell Signaling Technology), anti-t-ACC (1:500 or 1:250; Cell Signaling Technology), and anti-β-actin (1:2000; Fujifilm Wako Pure Chemicals, Osaka, Japan).

### Isolation of rat MVs and VICs

MVs were isolated from six-week-old male Wistar rats (CLEA Japan, Tokyo, Japan). The rats were kept under environmental controlled conditions (12-h light/dark cycle; 20 ℃−26 ℃ and 40%−60% humidity) and provided standard rodent chow (CE2; CLEA Japan) and water *ad libitum*. After euthanizing the rats via intraperitoneal administration of pentobarbital sodium (≥100 mg/kg), their MVs and tricuspid valves were harvested. *Ex vivo*, the MVs were used for experiments in organ culture, as described below. For the isolation of rat VICs (rVICs), valvular tissues were digested with type II collagenase (425 U/mL; Worthington Biochemical, Lakewood, NJ, USA) at 37 ℃ for 2 h. After washing with sterile phosphate-buffered saline, the cells were seeded into dishes and cultured in Dulbecco’s modified Eagle’s medium (DMEM) supplemented with 2% fetal bovine serum, 1% penicillin–streptomycin, rat recombinant fibroblast growth factor-basic (10 ng/mL; ProSpec, Ness-Ziona, Israel), and human recombinant insulin (50 ng/mL; Cell Science and Technology Institute, Miyagi, Japan) at 37 ℃ with 5% CO_2_. Cells at passages 3–5 were used for subsequent experiments.

### MV organ culture

After washing with phosphate-buffered saline, isolated MVs were soaked in DMEM supplemented with 0.5% fetal bovine serum at 37 °C with 5% CO_2_. The MVs were exposed to TGF-β (10 ng/mL) for 24 h or 72 h in the presence or absence of DAPA (100 μM; pre-treatment for 24 h). For histological analysis, MVs were fixed in 4% phosphate-buffered paraformaldehyde for 15–30 min. Protein was extracted using a radioimmunoprecipitation assay lysis buffer (Nacalai Tesque) for western blot analysis.

### Cell treatment

After reaching 70%–90% confluency, rVICs were subjected to serum starvation for 6 h and exposed to DAPA (100 μM) alone or TGF-β (10 ng/mL) in the presence or absence of DAPA (3, 10, 30, and 100 μM; pre-treatment for 24 h). Cell images were captured using a phase-contrast microscope (AXJ-5350TPH; WRAYMER, Osaka, Japan) equipped with a digital camera (TC-II plus; BioTools, Gunma, Japan). Protein lysates were obtained by homogenizing rVICs using a radioimmunoprecipitation assay lysis buffer.

### Small interfering RNA transfection

After reaching 70%–80% confluency, rVICs were transfected with small interfering RNA (siRNA) against AMPKα (5′-AGCCAAAGAUUUCUACUUGGCAACA-3′ and 5′-UGUUGCCAAGUAGAAAUCUUUGGCUUC-3′, final concentration: 5 nM; Integrated DNA Technologies, Coralville, IA, USA) or non-silencing siRNA (Integrated DNA Technologies). Six hours after transfection, cells were treated with DAPA (100 μM) for 24 h, followed by stimulation with TGF-β (10 ng/mL) for 30 min. The protein samples were subjected to western blot analyses.

### Cytotoxicity evaluation via lactate dehydrogenase assay

Next, DAPA cytotoxicity in rVICs was assessed by measuring lactate dehydrogenase activity using the Cytotoxicity Detection Kit (Dojindo, Kumamoto, Japan), according to the manufacturer’s instructions. Briefly, rVICs were seeded into a 96-well plate at 1.5 × 10^4^ cells/well and stimulated with DAPA for 24 h. After incubation with the working solution for 30 min, absorbance at 490 nm was measured using a microplate reader (Infinite 200 Pro; Tecan, Männedorf, Switzerland). Triton-X was used as a positive control.

### Western blotting

Protein concentration was determined using the bicinchoninic acid assay (Thermo Fisher Scientific, Waltham, MA, USA). Equal amounts of proteins (4–8 μg) were separated via sodium dodecyl sulfate-polyacrylamide gel electrophoresis (8% or 10%) and transferred onto nitrocellulose membranes (Pall, Port Washington, NY, USA). After blocking with 3% bovine serum albumin for 1 h, the membranes were incubated with primary antibodies at 4 ℃ overnight, and this was followed by incubation with horseradish peroxidase-conjugated secondary antibodies (1:10,000 dilution; 45 min; Cell Signaling Technology) and chemiluminescent substrates (enhanced chemiluminescent western blotting substrate; Promega, Madison, WI, USA or Chemi-Lumi One Ultra; Nacalai Tesque). Equal loading of proteins was confirmed by measuring the expression levels of each protein and β-actin. The primary and secondary antibodies were stripped using 0.1 mol/L glycine solution (pH 2.9). Phosphorylated and total protein bands were obtained from the same membrane. The results were analyzed using ImageJ software (v.1.50; NIH, Bethesda, MD, USA).

### Collagen gel contraction assay

Collagen gel solution was prepared on ice by mixing Cellmatrix Type I-A (type I collagen derived from porcine tendon; Nitta Gelatin, Osaka, Japan), 10 × MEM (Nitta Gelatin), and reconstituting buffer (Nitta Gelatin) in a ratio of 8:1:1. The final collagen concentration was 1.0 mg/mL. Then, rVICs (5.0 × 10^4^ cells/well) were centrifuged at 327 × *g* for 3 min at 4 ℃ and resuspended in the collagen gel solution. Subsequently, 400 μL of this suspension was aliquoted into a 24-well culture plate and allowed to polymerize at 37 ℃ for 30 min. Cell-containing collagen gel was cultured overnight in DMEM supplemented with 0.5% fetal bovine serum, and the gel was then released from the wells and pre-treated with DAPA for 24 h. After culturing the gel with TGF-β for 24 h, the area of each gel was measured using ImageJ software.

### Histological analysis

Formalin-fixed MVs were paraffinized and sliced into thin sections. (5-μm thickness). For histological analysis, thin paraffin sections were stained with hematoxylin and eosin or picrosirius red and alcian blue. For picrosirius red and alcian blue staining, deparaffinized sections were stained with alcian blue solution (Fujifilm Wako Pure Chemicals) for 45 min. After staining with picrosirius red solution (picric acid: 1.25 g, Sirius red: 0.1 g, and distilled water: 100 mL) for 15 min, the sections were immersed in 0.5% acetic acid solution for 5 min. Using a hematoxylin and eosin-stained section, the MV leaflet thickness was measured. Layer structure and accumulation of acid mucopolysaccharide in the MV were assessed via morphological observation of Sirius red-positive collagen and calculation of the alcian blue-positive area to valve area ratio, respectively. These measurements were performed using ImageJ software.

### Statistical analyses

Statistical analyses were performed using GraphPad Prism software (v.8.4.3; GraphPad Software, San Diego, CA, USA). One-way analysis of variance followed by Tukey’s post-hoc test was used to assess the differences among groups. All data are calculated from different biological replicates and presented as mean ± standard deviation. Statistical significance was set at *p* < 0.05.

## Results

### DAPA exerts no cytotoxic effects on rVICs

**Fig. 1:**
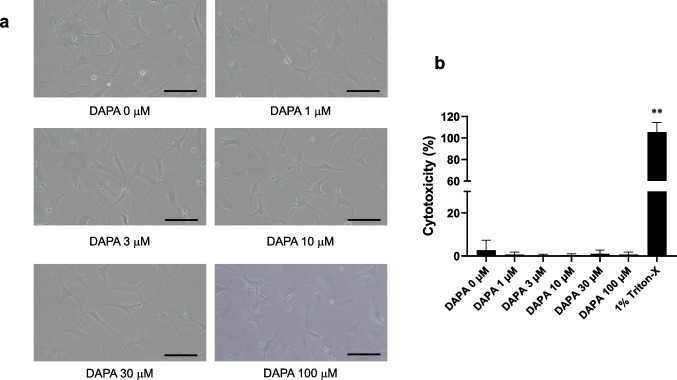
Cytotoxic effects of dapagliflozin (DAPA) on rat valvular interstitial cells (rVICs). After treatment with DAPA (1, 3, 10, 30, and 100 μM) for 24 h, cell morphology was observed, and rVIC cytotoxicity was assessed using the lactate dehydrogenase assay. (**a**) Representative photomicrographs of rVICs. Morphological observation was performed using five independent cells. Scale bar = 100 μm. (**b**) Lactate dehydrogenase assay results are expressed as the percentage of cytotoxicity (n = 3). Triton-X (1%) was used as a positive control. Data are presented as mean ± standard deviation (SD), with “n” denoting the number of independent experiments using cells from a different stock or passage. **p < 0.01 vs. control

We investigated the effects of various concentrations of DAPA (1, 3, 10, 30, and 100 μM) on rVIC viability. Notably, rVIC viability and morphology were not affected by DAPA (n = 5; Fig. 1a). Lactate dehydrogenase assay results indicated that DAPA did not exert cytotoxic effects on rVICs (n = 3; Fig. 1b). Therefore, 3, 10, 30, and 100 μM DAPA were selected for subsequent experiments.

### DAPA inhibits TGF-β-induced α-SMA expression and collagen gel contraction in rVICs

**Fig. 2:**
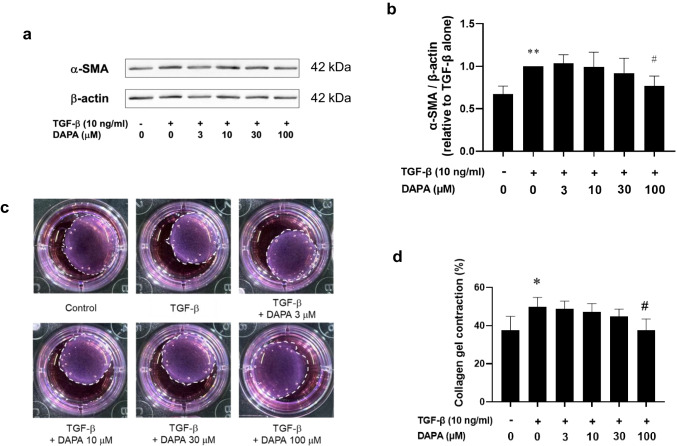
Effect of DAPA on transforming growth factor (TGF)-β-induced alpha-smooth muscle actin (α-SMA) expression and collagen gel contraction in rVICs. The rVICs were treated with TGF-β (10 ng/mL) for 16 h or 24 h in the presence or absence of DAPA (3, 10, 30, and 100 μM; pre-treatment for 24 h). The α-SMA expression levels were determined via western blotting (n = 7) and normalized to that of β-actin. Representative blot images are shown (**a**). Cellular contractile forces generated by VICs were measured using a collagen gel contraction assay (n = 5). Representative images of the collagen gel are presented (**c**). Results are expressed as the fold increase relative to that of TGF-β alone (**b**) or the percentage of contraction (**d**). Data are presented as mean ± SD, with “n” denoting the number of independent experiments using cells from a different stock or passage. *p < 0.05 vs. control; **p < 0.01 vs. control; #*p* < 0.05 vs. TGF-β alone

We investigated whether DAPA affects the TGF-β-induced increase in α-SMA expression levels in rVICs. The α-SMA expression levels were significantly increased by TGF-β (control: 0.67 ± 0.09-fold increase relative to that with TGF-β alone; *p* < 0.05; n = 7; Fig. 2a, b). However, DAPA significantly suppressed this effect (TGF-β + DAPA [100 μM]: 0.77 ± 0.12-fold increase relative to that with TGF-β alone; *p* < 0.05; n = 7; Fig. 2a, b). Cellular contraction is increased in differentiated α-SMA-positive VICs. A collagen gel contraction assay was thus used to assess the contractile functions of rVICs. The contraction was significantly increased by TGF-β (control: 37.7 ± 7.2% vs. TGF-β alone: 49.9 ± 5.0%; *p* < 0.05; n = 5; Fig. 2c, d), and DAPA significantly suppressed this effect (TGF-β + DAPA [100 μM]: 37.6 ± 5.9%; *p* < 0.05; n = 5; Fig. 2c, d).

### DAPA inhibits TGF-β-induced Smad2 and Smad3 phosphorylation via AMPKα activation in rVICs

**Fig. 3:**
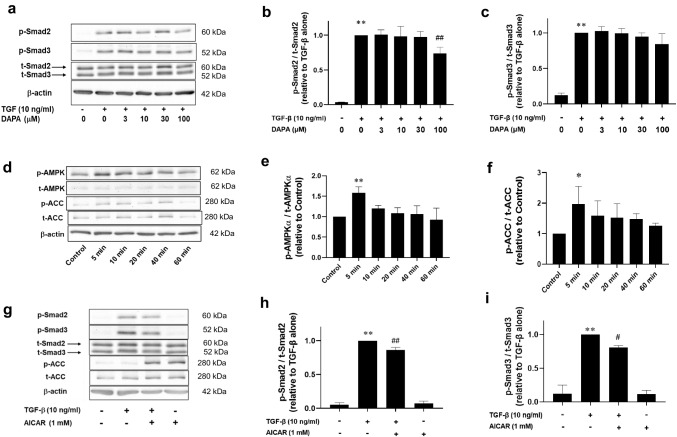

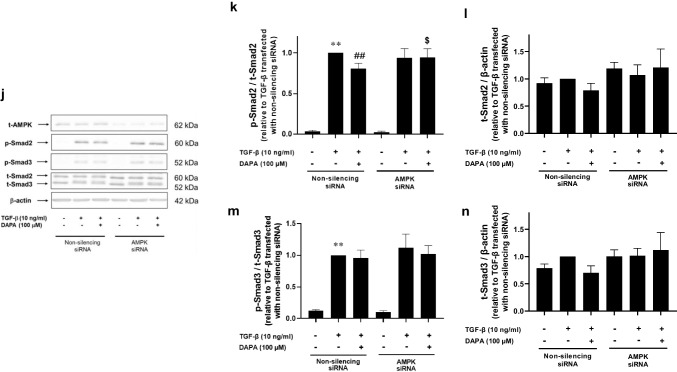
Effect of DAPA on TGF-β-induced Smad phosphorylation and adenosine monophosphate-activated protein kinase (AMPK)α activation in rVICs. The rVICs were treated with DAPA (100 μM) alone for 5–60 min (n = 4; d–f), or DAPA (3, 10, 30, and 100 μM, n = 4; a–c) or the AMPK activator 5-aminoimidazole-4-carboxamide 1-β-d-ribofuranoside (AICAR; 1 mM, n = 4; g–i) for 24 h followed by treatment with TGF-β (10 ng/mL) for 30 min. For gene knockdown, cells were transfected with small interfering RNA (siRNA) targeting AMPKα (5 nM) 6 h before the DAPA and TGF-β stimulation (n = 5; j–n). Phosphorylation levels of Smad2, Smad3, AMPKα, and acetyl-CoA carboxylase (ACC) were determined using western blotting. Phosphorylated protein levels were normalized to the total protein levels. Representative blot images are shown (**a**, **d**, **g**, and **j**). Results are expressed as the fold increase relative to that in the control (**e** and **f**), that with TGF-β alone (**b**, **c**, **h**, and **i**), or non-silencing siRNA-transfected cells treated with TGF-β alone (k–n). Data are presented as mean ± SD, with “n” denoting the number of independent experiments using cells from a different stock or passage. *p < 0.05 vs. control; **p < 0.01 vs. control or control with non-silencing siRNA; #*p* < 0.05 vs. TGF-β alone; ##*p* < 0.01 vs. TGF-β alone or TGF-β with non silencing siRNA; $*p* < 0.05 vs. TGF-β + DAPA (100 μM) with non-silencing siRNA

We examined the effects of DAPA on the TGF-β-induced phosphorylation of Smad2 and Smad3, positive regulators of α-SMA expression in rVICs. Protein expression levels of p-Smad2 (control: 0.033 ± 0.005-fold increase relative to that with TGF-β alone; *p* < 0.01; n = 4; Fig. 3a, b) and p-Smad3 (control: 0.12 ± 0.03-fold increase relative to that with TGF-β alone; *p* < 0.01; n = 4; Fig. 3a, c) were significantly increased by TGF-β. However, DAPA significantly suppressed the increased expression of p-Smad2 (TGF-β + DAPA [100 μM]: 0.74 ± 0.09-fold increase relative to that with TGF-β alone; *p* < 0.01; n = 4; Fig. 3a, b) and tended to decrease the increased p-Smad3 levels, but this change was not statistically significant (TGF-β + DAPA [100 μM]: 0.84 ± 0.15-fold increase relative to that with TGF-β alone; *p* = 0.07; n = 4; Fig. 3a, c).

AMPKα activation inhibits the TGF-β/Smad signaling pathway, and DAPA activates AMPKα in H9c2 cardiomyoblasts [13]. Thus, we investigated whether DAPA increases AMPKα activity in rVICs. Protein expression of p-AMPKα was significantly increased by DAPA (100 μM; 5 min: 1.58 ± 0.15-fold increase relative to that in the control; *p* < 0.01; n = 4; Fig. 3d, e). Additionally, protein expression of p-ACC, an AMPK substrate, was significantly increased by DAPA (100 μM; 5 min: 1.97 ± 0.58-fold increase relative to that in the control; *p* < 0.05; n = 4; Fig. 3d, f).

Next, we examined the effect of the AMPK agonist AICAR on the TGF-β-induced phosphorylation of Smad2 and Smad3. Protein expression levels of p-Smad2 (control: 0.05 ± 0.03-fold increase relative to that with TGF-β alone; *p* < 0.01; n = 4; Fig. 3g, h) and p-Smad3 (control: 0.12 ± 0.13-fold increase relative to that with TGF-β alone; *p* < 0.01; n = 4; Fig. 3g, i) were significantly increased by TGF-β. However, AICAR significantly suppressed the increase in expression levels of p-Smad2 (TGF-β + AICAR: 0.86 ± 0.04-fold increase relative to that with TGF-β alone; *p* < 0.01; n = 4; Fig. 3g, h) and p-Smad3 (TGF-β + AICAR: 0.81 ± 0.03-fold increase relative to that with TGF-β alone; *p* < 0.05; n = 4; Fig. 3g, i).

In addition, we investigated whether AMPKα gene knockdown reverses the inhibitory effect DAPA exerts on TGF-β-induced Smad phosphorylation. TGF-β increased Smad2 phosphorylation (control with non-silencing siRNA: 0.04 ± 0.01-fold increase relative to TGF-β with non-silencing siRNA; *p* < 0.01; n = 5; Fig. 3j–l), whereas DAPA significantly inhibited it (TGF-β + DAPA [100 μM] with non-silencing siRNA: 0.81 ± 0.06-fold increase relative to TGF-β with non-silencing siRNA; *p* < 0.01; n = 5; Fig. 3j–l) in non-silencing siRNA-transfected cells. AMPKα gene knockdown impeded the inhibitory effect of DAPA (TGF-β + DAPA [100 μM] with AMPK siRNA: 0.95 ± 0.11-fold increase relative to TGF-β with non-silencing siRNA; *p* < 0.05 vs. TGF-β + DAPA [100 μM] with non-silencing siRNA; n = 5; Fig. 3j–l). Since DAPA slightly reduced the expression levels of p-Smad3 and t-Smad3 in non-silencing siRNA-transfected cells, the p-Smad3 to t-Smad3 ratio was similar to that after treatment with TGF-β alone (TGF-β + DAPA [100 μM] with non-silencing siRNA: 0.95 ± 0.13-fold increase relative to TGF-β with non-silencing siRNA; n = 5; Fig. 3j, m, n).

### DAPA inhibits TGF-β-induced thickening and myxomatous degeneration of isolated rat MV

**Fig. 4:**
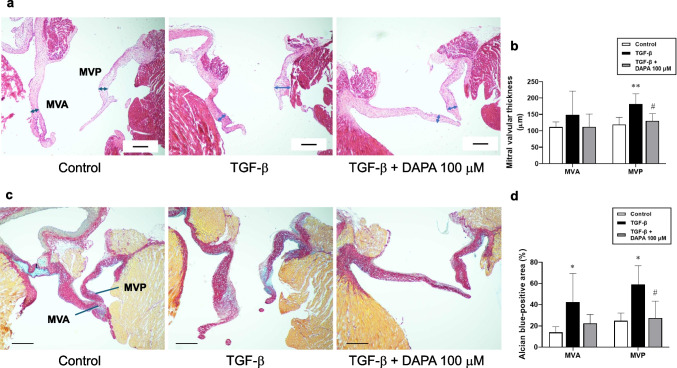
Effects of DAPA on TGF-β-induced mitral valvular thickening and degeneration in rats. After isolation, the rat mitral valve (MV) was treated with DAPA (100 μM) for 24 h followed by treatment with TGF-β (10 ng/mL) for 72 h. Paraffin sections were subsequently prepared. Representative hematoxylin and eosin (n = 5; **a**)- and picrosirius red/alcian blue (n = 5; **c**)-stained sections are shown. Scale bar = 200 μm. Double-headed arrow indicates the thickness of MV leaflets. The thickness (n = 5; **b**) or alcian blue-positive area to valve area ratio (n = 5; **d**) of the anterior and posterior leaflet of the MV is expressed as a bar graph. Data are presented as mean ± SD, with “n” denoting the number of MV tissues isolated from a different rat. MVA: anterior MV leaflet; MVP: posterior MV leaflet. *p < 0.05 vs. control; **p < 0.01 vs. control; #*p* < 0.05 vs. TGF-β alone

Differentiated VICs can induce MV thickening in accordance with myxomatous degeneration. Therefore, we investigated the effects of DAPA on TGF-β-induced histopathological changes in rat MV tissues. TGF-β significantly increased MV posterior leaflet thickness (anterior leaflet; control: 111.6 ± 15.8 μm vs. TGF-β alone: 148.2 ± 73.0 μm; *p* = 0.48; n = 5, posterior leaflet; control: 119.4 ± 21.6 μm vs. TGF-β alone: 181.9 ± 30.7 μm; *p* < 0.01; n = 5; Fig. 4a, b), whereas DAPA significantly reduced it (anterior leaflet; TGF-β + DAPA [100 μM]: 112.4 ± 38.6 μm; *p* = 0.49; n = 5, posterior leaflet; TGF-β + DAPA [100 μM]: 130.6 ± 22.0 μm; *p* < 0.05; n = 5; Fig. 4a, b). In addition, TGF-β induced destruction of the layer structure and significantly increased the accumulation of acid mucopolysaccharides (alcian blue-positive area; anterior leaflet; control: 13.92 ± 5.46% vs. TGF-β alone: 40.63 ± 23.79%; *p* < 0.05; n = 5, posterior leaflet; control: 24.93 ± 7.08% vs. TGF-β alone: 54.12 ± 18.90%; *p* < 0.05; n = 5; Fig. 4c, d) in rat MV. However, DAPA partly suppressed the structural destruction and acid mucopolysaccharide accumulation (alcian blue-positive area; anterior leaflet; TGF-β + DAPA [100 μM]: 22.38 ± 8.42%; *p* = 0.17; n = 5, posterior leaflet; TGF-β + DAPA [100 μM]: 27.49 ± 15.80%; *p* < 0.05; n = 5; Fig. 4c, d).

### DAPA inhibits TGF-β-induced α-SMA expression and Smad phosphorylation via AMPKα activation in isolated rat MV

**Fig. 5:**
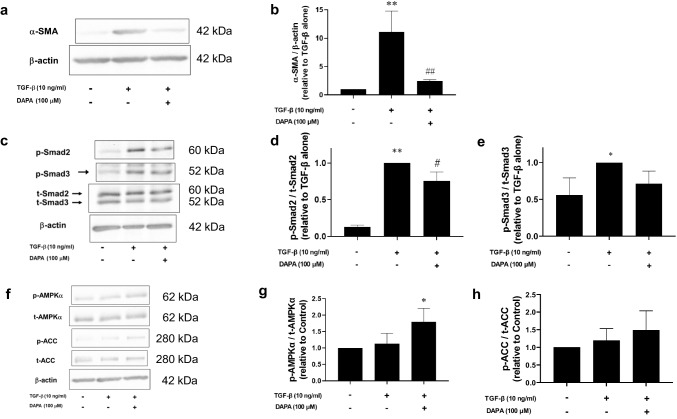
Effect of DAPA on TGF-β-induced activation of the Smad2/3/α-SMA pathway and AMPK phosphorylation in degenerated MVs of rats. After isolation, the rat MV was treated with DAPA (100 μM) for 24 h followed by treatment with TGF-β (10 ng/mL) for 72 h (n=3; **a**, **b**) or 24 h (n=3; **c**–**h**), and the proteins were then extracted. The expression levels of α-SMA, p-Smad2, p-Smad3, p-AMPKα and p-ACC were determined using western blotting. Representative blot images are shown (**a**, **c**, and **f**). The expression levels of α-SMA and phosphorylated proteins were normalized to those of β-actin and the total protein, respectively. Results are expressed as the fold increase relative to that with TGF-β alone (**b**, **d**, **e**, **g**, and **h**). Data are presented as mean ± SD, with “n” denoting the number of MV tissues isolated from a different rat. *p<0.05 vs. control; **p<0.01 vs. control; #*p*<0.05 vs. TGF-β alone; ##*p*<0.01 vs. TGF-β alone

Next, we examined the effects of DAPA on AMPKα activity and TGF-β-induced activation of the Smad/α-SMA signaling pathway in isolated rat MV. The α-SMA expression levels were significantly increased by TGF-β (TGF-β alone: 11.07 ± 3.69-fold increase relative to that in the control; *p* < 0.01; n = 3; Fig. 5a, b). DAPA significantly suppressed this effect (TGF-β + DAPA [100 μM]: 2.43 ± 0.24-fold increase relative to that in the control; *p* < 0.01; n = 3; Fig. 5a, b). TGF-β significantly increased the expression levels of p-Smad2 (control: 0.13 ± 0.03-fold increase relative to that with TGF-β alone; *p* < 0.01; n = 3; Fig. 5c, d) and p-Smad3 (control: 0.56 ± 0.23-fold increase relative to that with TGF-β alone; *p* < 0.05; n = 3; Fig. 5c, e), whereas DAPA significantly suppressed the phosphorylation of Smad2 (TGF-β + DAPA [100 μM]: 0.76 ± 0.12-fold increase relative to that with TGF-β alone; *p* < 0.05; n = 3; Fig. 5c, d), but the observed inhibition of Smad3 phosphorylation by DAPA was not significant (TGF-β + DAPA [100 μM]: 0.72 ± 0.17-fold increase relative to that with TGF-β alone; *p* = 0.17; n = 3; Fig. 5c, e). In addition, DAPA significantly phosphorylated AMPKα (TGF-β + DAPA [100 μM]: 1.80 ± 0.41-fold increase relative to that in the control; *p* < 0.05; n = 3; Fig. 5f, g), but the phosphorylation of ACC was weak (TGF-β + DAPA [100 μM]: 1.49 ± 0.54-fold increase relative to that in the control; *p* = 0.30; n = 3; Fig. 5f, h) in the presence of TGF-β.

## Discussion

In this study, DAPA inhibited TGF-β-induced increase in α-SMA expression (Fig. 2a, b), collagen gel contraction (Fig. 2c, d), and phosphorylation of Smad2 and Smad3 (Fig. 3a–c) in rVICs. DAPA inhibits the increase in α-SMA, TGF-β, Smad2, and Smad3 levels in the myocardial tissues of aortic constriction-induced heart failure model rabbits [12]. Additionally, DAPA inhibits endothelial–mesenchymal transition by inhibiting α-SMA expression in TGF-β1-stimulated human umbilical vein endothelial and mouse aortic endothelial cells [14]. These findings suggest that DAPA suppresses TGF-β-induced transdifferentiation of rVICs partly by inhibiting the Smad signaling pathway.

AMPK activation plays a protective role against cardiovascular diseases. Canagliflozin, an SGLT2 inhibitor, attenuates monocrotaline-induced pulmonary hypertension via the SGLT1/AMPK pathway in rats [15]. DAPA activates AMPKα thereby alleviating left ventricular remodeling and cardiac dysfunction in diabetic rats [11]. In the present study, DAPA increased AMPKα phosphorylation (Fig. 3d, e). Metformin, an AMPK activator, inhibits the TGF-β-induced increase in Smad2 and Smad3 phosphorylation in A549 lung adenocarcinoma cells [16]. Similarly, another AMPK activator AICAR inhibited TGF-β-induced increases in p-Smad2 and p-Smad3 levels in the present study (Fig. 3g–i). Furthermore, AMPKα gene knockdown impeded the inhibitory effect of DAPA on the TGF-β-induced phosphorylation of Smad2 (Fig. 3j–l). Therefore, DAPA induced AMPK activation, thereby inhibiting TGF-β-induced activation of the Smad pathway in rVICs.

**Fig. 6:**
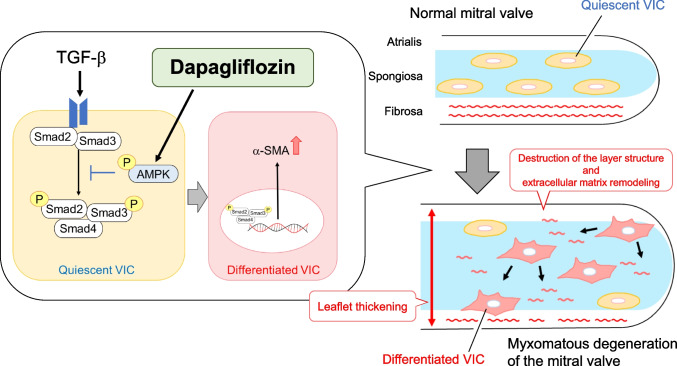
Summary of the present study results. Activation of AMPK by DAPA suppresses the TGF-β-induced transdifferentiation of quiescent VIC, partly by inhibiting the Smad signaling pathway, thereby alleviating MV leaflet thickening and extracellular matrix remodeling

TGF-β expression is increased in the MV of humans [17, 18] and dogs [19] with MMVD. Additionally, the expression levels of α-SMA, p-Smad2, and p-Smad3 are upregulated in degenerated MV [10]. Activated VICs are characterized by increased expression of α-SMA and contractile myofibroblast-like phenotype [5]. Although quiescent VICs are mainly observed in a healthy MV, the number of activated VICs is increased in humans [10, 18] and dogs [9] with MMVD. In the present study, DAPA inhibited the TGF-β-induced increase in α-SMA levels and Smad2 and Smad3 phosphorylation levels in isolated rat MV (Fig. 5a–e). Additionally, DAPA inhibited TGF-β-induced MV thickening and acid mucopolysaccharide accumulation (Fig. 4). These results suggest that DAPA suppresses MV degeneration by inhibiting the TGF-β/Smad-mediated activation of VICs in MMVD (Fig. 6).

This study had several limitations. First, DAPA and AMPK agonists did not completely inhibit Smad phosphorylation in rVICs. Smad-independent pathways also regulate α-SMA expression in TGF-β signaling. The PI3K/Akt and p38 MAPK signaling pathways are activated in the diseased MV and VICs [20, 21]. In NIH 3T3 fibroblasts, constitutive activation of Akt1 enhances TGF-β-induced α-SMA expression; conversely, dominant negative Akt1 reduces it [22]. TGF-β-induced α-SMA expression is inhibited by a p38 MAPK inhibitor SB203580 in porcine proximal tubular epithelial cells [23]. TGF-β also increases α-SMA expression by activating the RhoA/Rho-associated protein kinase (ROCK) signaling pathway in rat kidney fibroblast cells. RhoA gene knockdown or pharmacological inhibition of ROCK suppresses TGF-β-induced α-SMA expression [24]. Empagliflozin inhibits phosphorylation of myosin phosphatase target subunit 1, a substrate of ROCK, in the myocardium of db/db diabetic mice [25]. These findings suggest that DAPA has the potential to suppress TGF-β-induced increases in α-SMA expression and VIC activation by inhibiting signaling pathways other than the Smad-dependent pathways. Second, while the sample size was determined based on similar published reports, for which statistical differences were demonstrated [9, 10, 26], the use of more samples might have increased the statistical power for detecting the effects of DAPA. Third, as the present in vitro and ex vivo study remains at an early preclinical stage, further in vivo validation using MMVD animal models is required for the future clinical extrapolation to canine or human patients with MMVD. We thus plan to investigate the inhibitory effects of DAPA on the progressive MV degeneration of spontaneous MMVD model, FVB mice [27]. Further studies to explore the detailed inhibitory mechanisms of DAPA are warranted. Prophylactic therapy with canine gonadal tissue-derived mesenchymal stem cells delayed MMVD progression in client-owned dogs [28]. Similarly, long-term effects of DAPA on MMVD progression need to be investigated in the future.

In conclusion, DAPA reduces MV degeneration and thickening by inhibiting the transdifferentiation of quiescent VICs to their active state, thereby potentially serving as a novel therapeutic agent for humans and dogs with MMVD.

## Supplementary Information

Below is the link to the electronic supplementary material.

ESM1 (PPTX 90.4 MB)

ESM2 (PPTX 111 MB)

ESM3 (PPTX 140 MB)

ESM4 (PPTX 166 MB)

ESM5 (PPTX 80.6 MB)

ESM6 (PPTX 106 MB)

## Data Availability

Detailed data for this study are available from the corresponding author upon reasonable request.
