# Supplementary material for: Dapagliflozin inhibits TGF-β-induced transdifferentiation of valvular interstitial cells and mitral valvular degeneration
Source: J Mol Med (Berl). 2025 Dec 15;104(1):4. doi: 10.1007/s00109-025-02615-z (PMC12702807; doi:10.1007/s00109-025-02615-z)
Supplement: Supplementary file 1 — (PPTX 90.4 MB) [file 109_2025_2615_MOESM1_ESM.pptx]

## Slide 1
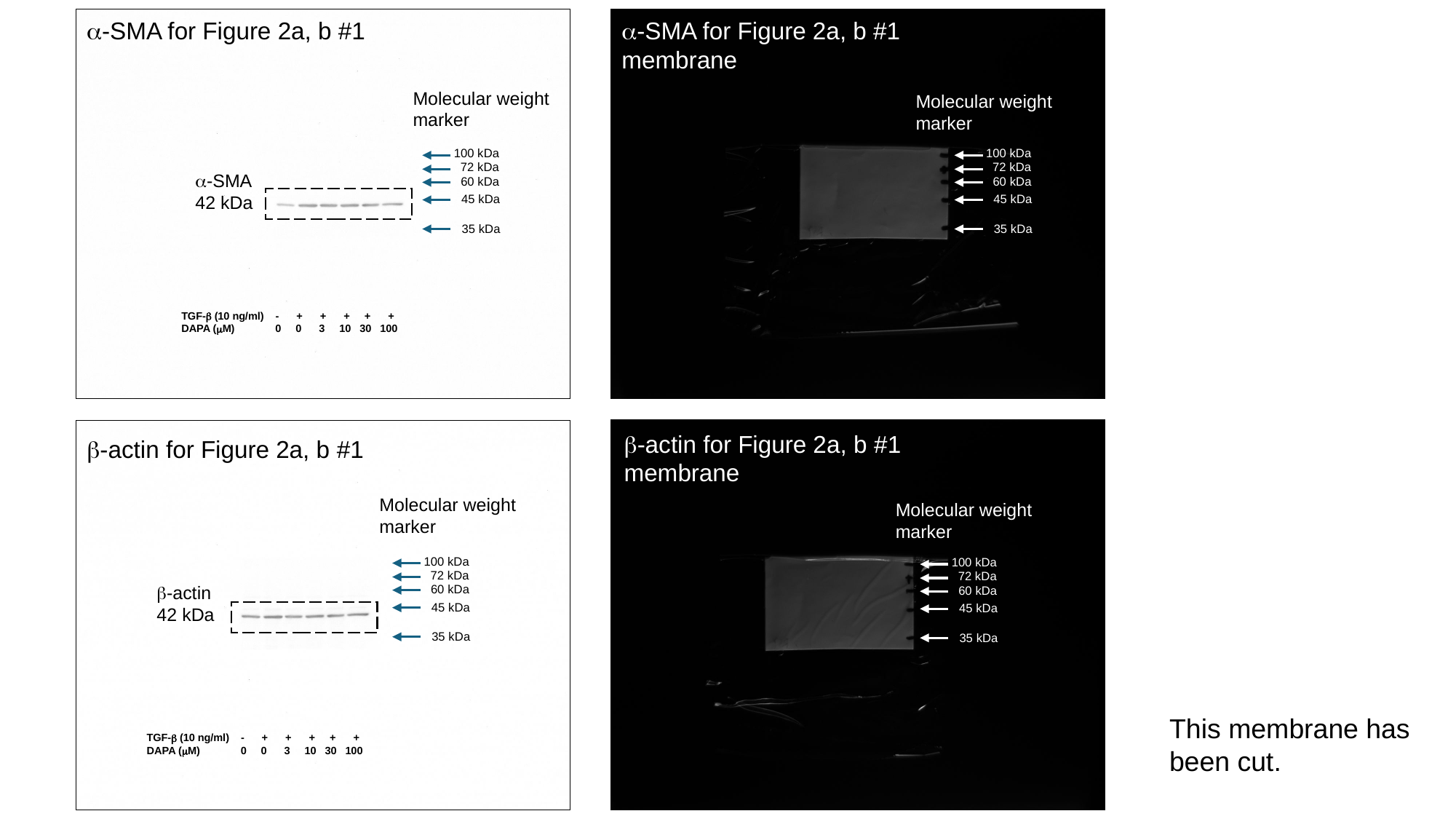

a-SMA for Figure 2a, b #1
a-SMA for Figure 2a, b #1 membrane
Molecular weight
marker
Molecular weight
marker
100 kDa
72 kDa
60 kDa
45 kDa
35 kDa
100 kDa
72 kDa
60 kDa
45 kDa
35 kDa
a-SMA
42 kDa
TGF-b (10 ng/ml) - + + + + +
DAPA (mM) 0 0 3 10 30 100
b-actin for Figure 2a, b #1 membrane
b-actin for Figure 2a, b #1
Molecular weight
marker
Molecular weight
marker
100 kDa
72 kDa
60 kDa
45 kDa
35 kDa
100 kDa
72 kDa
60 kDa
45 kDa
35 kDa
b-actin
42 kDa
This membrane has been cut.
TGF-b (10 ng/ml) - + + + + +
DAPA (mM) 0 0 3 10 30 100

## Slide 2
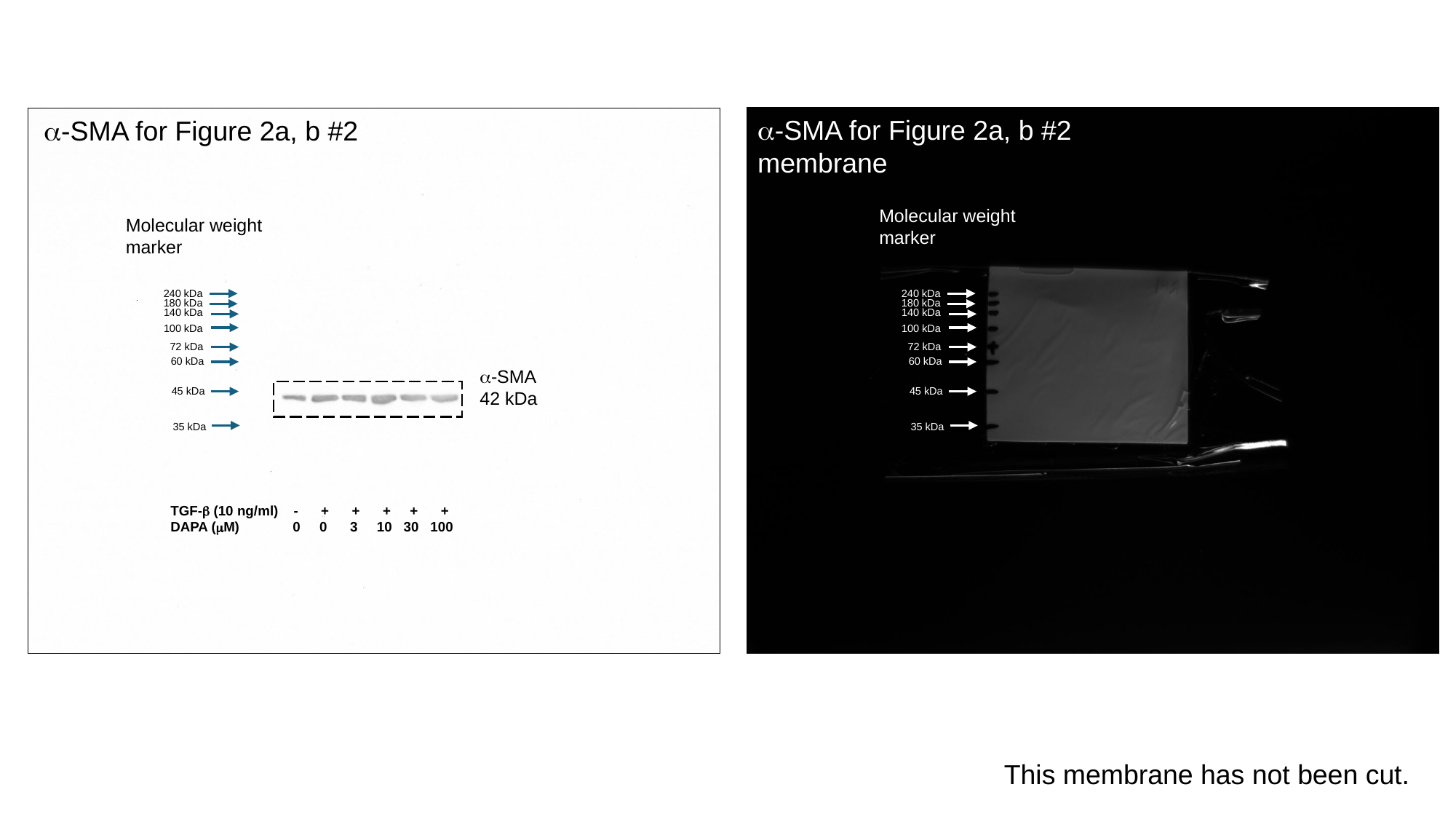

a-SMA for Figure 2a, b #2 membrane
a-SMA for Figure 2a, b #2
Molecular weight
marker
Molecular weight
marker
240 kDa
180 kDa
140 kDa
100 kDa
72 kDa
60 kDa
45 kDa
35 kDa
240 kDa
180 kDa
140 kDa
100 kDa
72 kDa
60 kDa
45 kDa
35 kDa
a-SMA
42 kDa
TGF-b (10 ng/ml) - + + + + +
DAPA (mM) 0 0 3 10 30 100
This membrane has not been cut.

## Slide 3
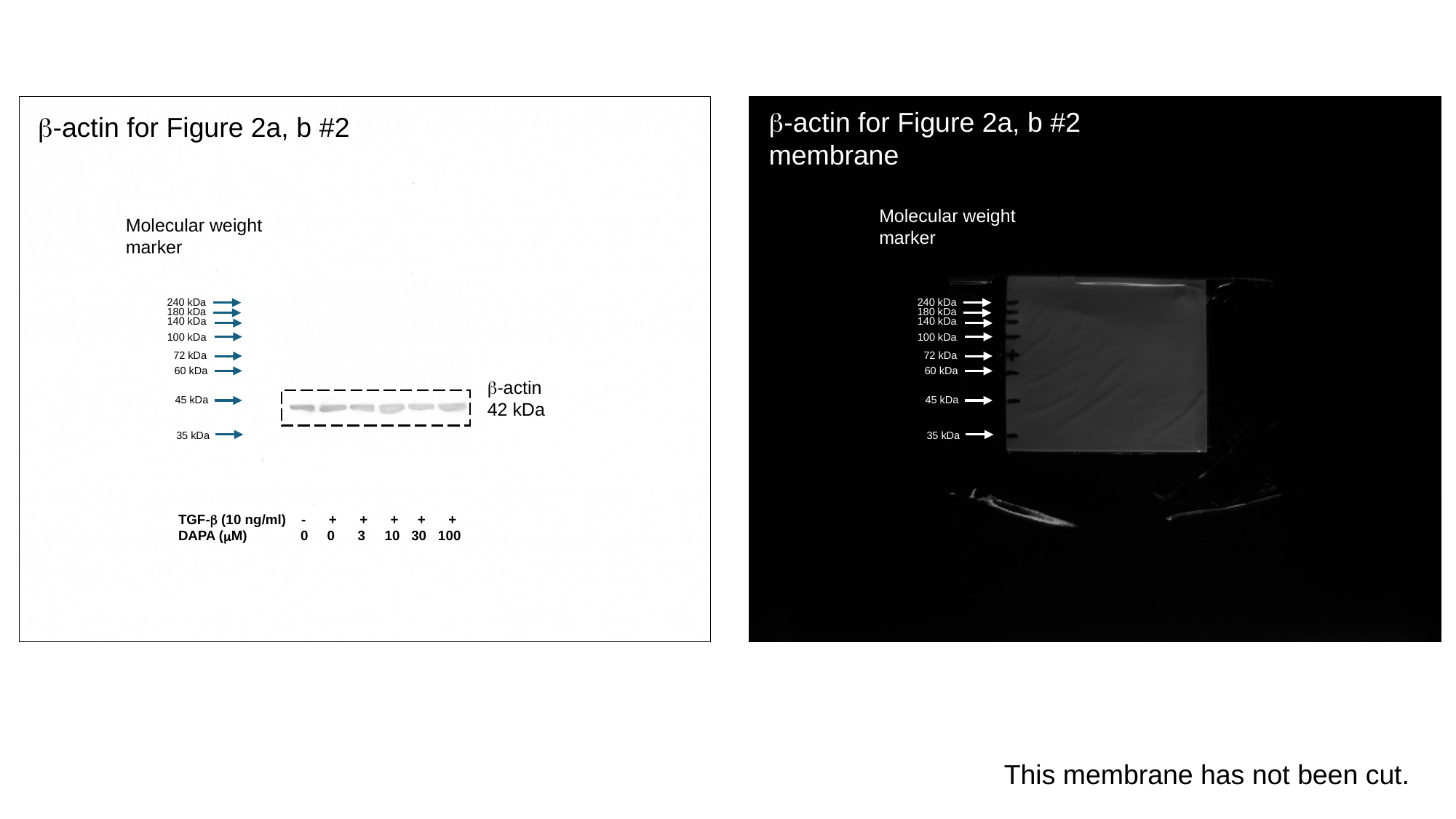

b-actin for Figure 2a, b #2 membrane
b-actin for Figure 2a, b #2
Molecular weight
marker
Molecular weight
marker
240 kDa
180 kDa
140 kDa
100 kDa
72 kDa
60 kDa
45 kDa
35 kDa
240 kDa
180 kDa
140 kDa
100 kDa
72 kDa
60 kDa
45 kDa
35 kDa
b-actin
42 kDa
TGF-b (10 ng/ml) - + + + + +
DAPA (mM) 0 0 3 10 30 100
This membrane has not been cut.

## Slide 4
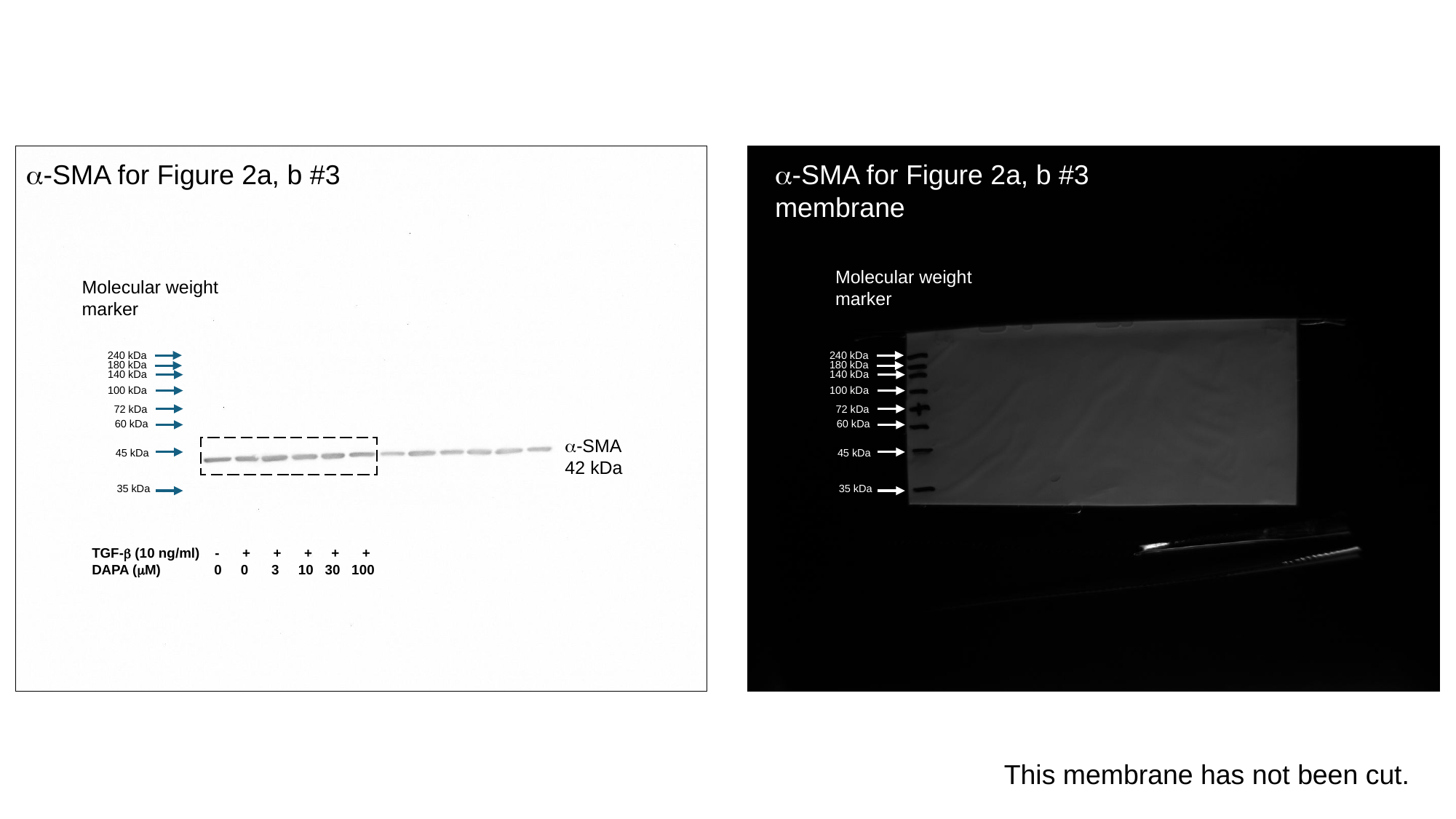

a-SMA for Figure 2a, b #3
a-SMA for Figure 2a, b #3 membrane
Molecular weight
marker
Molecular weight
marker
240 kDa
180 kDa
140 kDa
100 kDa
72 kDa
60 kDa
45 kDa
35 kDa
240 kDa
180 kDa
140 kDa
100 kDa
72 kDa
60 kDa
45 kDa
35 kDa
a-SMA
42 kDa
TGF-b (10 ng/ml) - + + + + +
DAPA (mM) 0 0 3 10 30 100
This membrane has not been cut.

## Slide 5
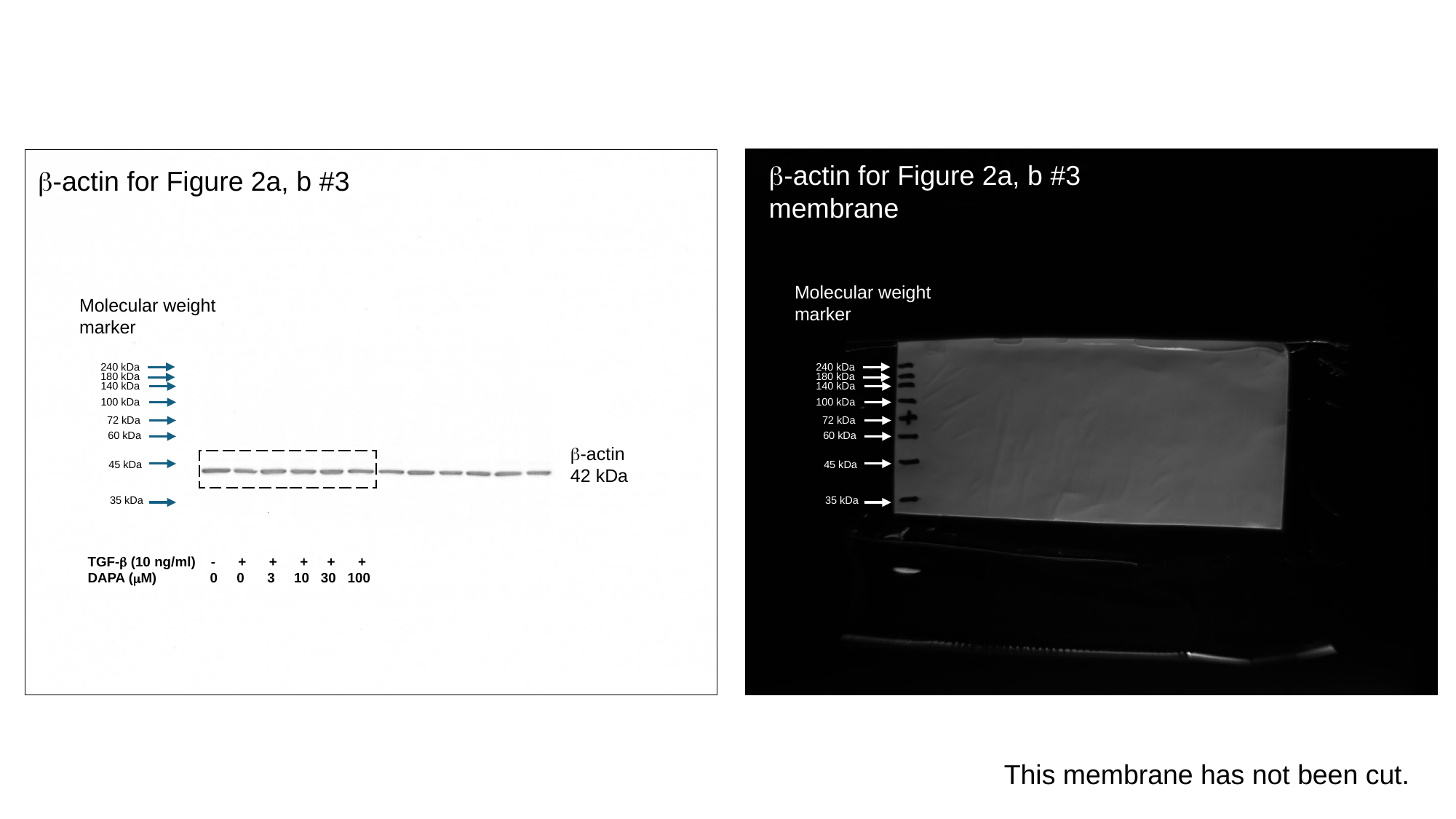

b-actin for Figure 2a, b #3 membrane
b-actin for Figure 2a, b #3
Molecular weight
marker
Molecular weight
marker
240 kDa
180 kDa
140 kDa
100 kDa
72 kDa
60 kDa
45 kDa
35 kDa
240 kDa
180 kDa
140 kDa
100 kDa
72 kDa
60 kDa
45 kDa
35 kDa
b-actin
42 kDa
TGF-b (10 ng/ml) - + + + + +
DAPA (mM) 0 0 3 10 30 100
This membrane has not been cut.

## Slide 6
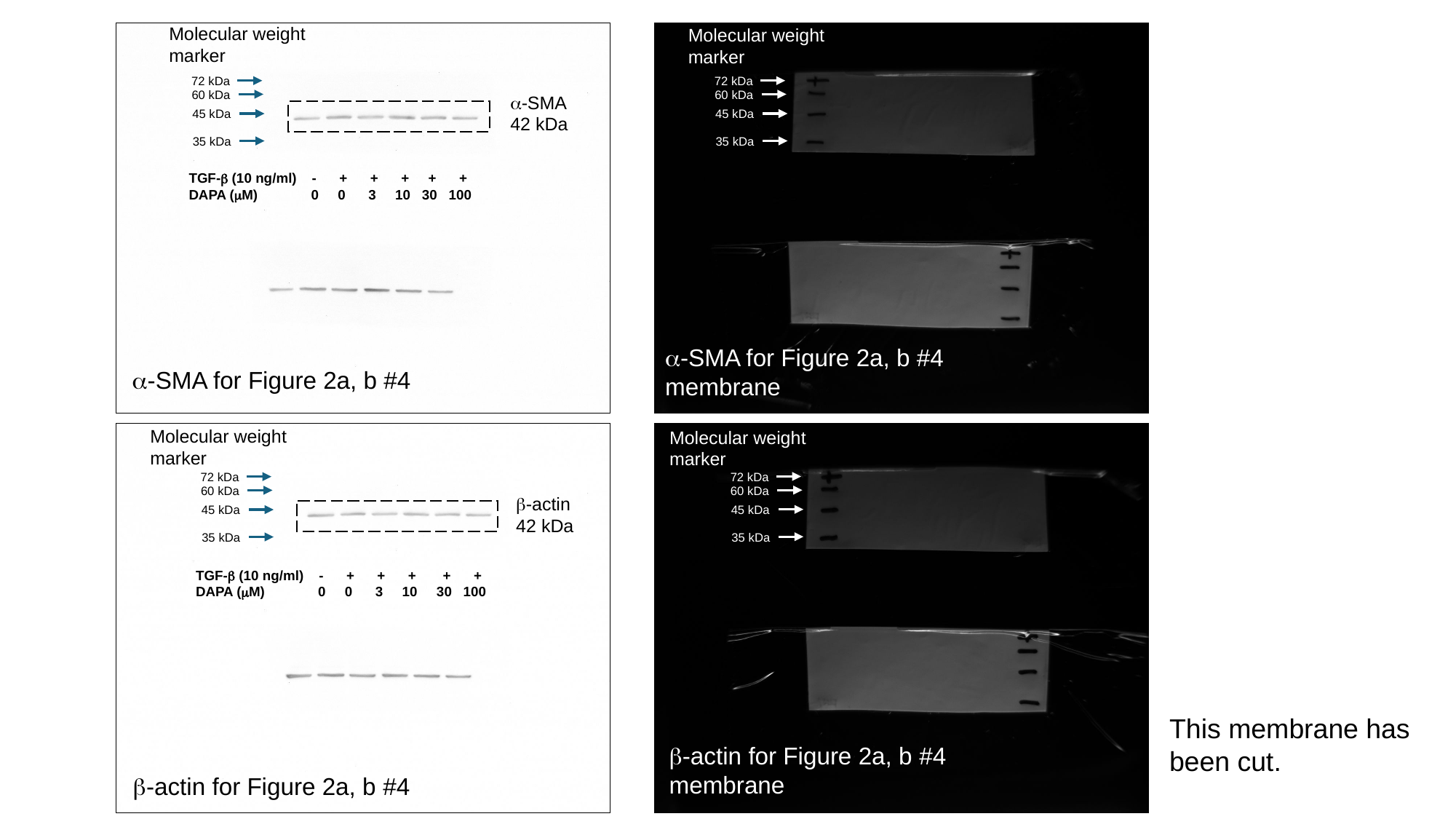

Molecular weight
marker
Molecular weight
marker
72 kDa
60 kDa
45 kDa
35 kDa
72 kDa
60 kDa
45 kDa
35 kDa
a-SMA
42 kDa
TGF-b (10 ng/ml) - + + + + +
DAPA (mM) 0 0 3 10 30 100
a-SMA for Figure 2a, b #4 membrane
a-SMA for Figure 2a, b #4
Molecular weight
marker
Molecular weight
marker
72 kDa
60 kDa
45 kDa
35 kDa
72 kDa
60 kDa
45 kDa
35 kDa
b-actin
42 kDa
TGF-b (10 ng/ml) - + + + + +
DAPA (mM) 0 0 3 10 30 100
This membrane has been cut.
b-actin for Figure 2a, b #4 membrane
b-actin for Figure 2a, b #4

## Slide 7
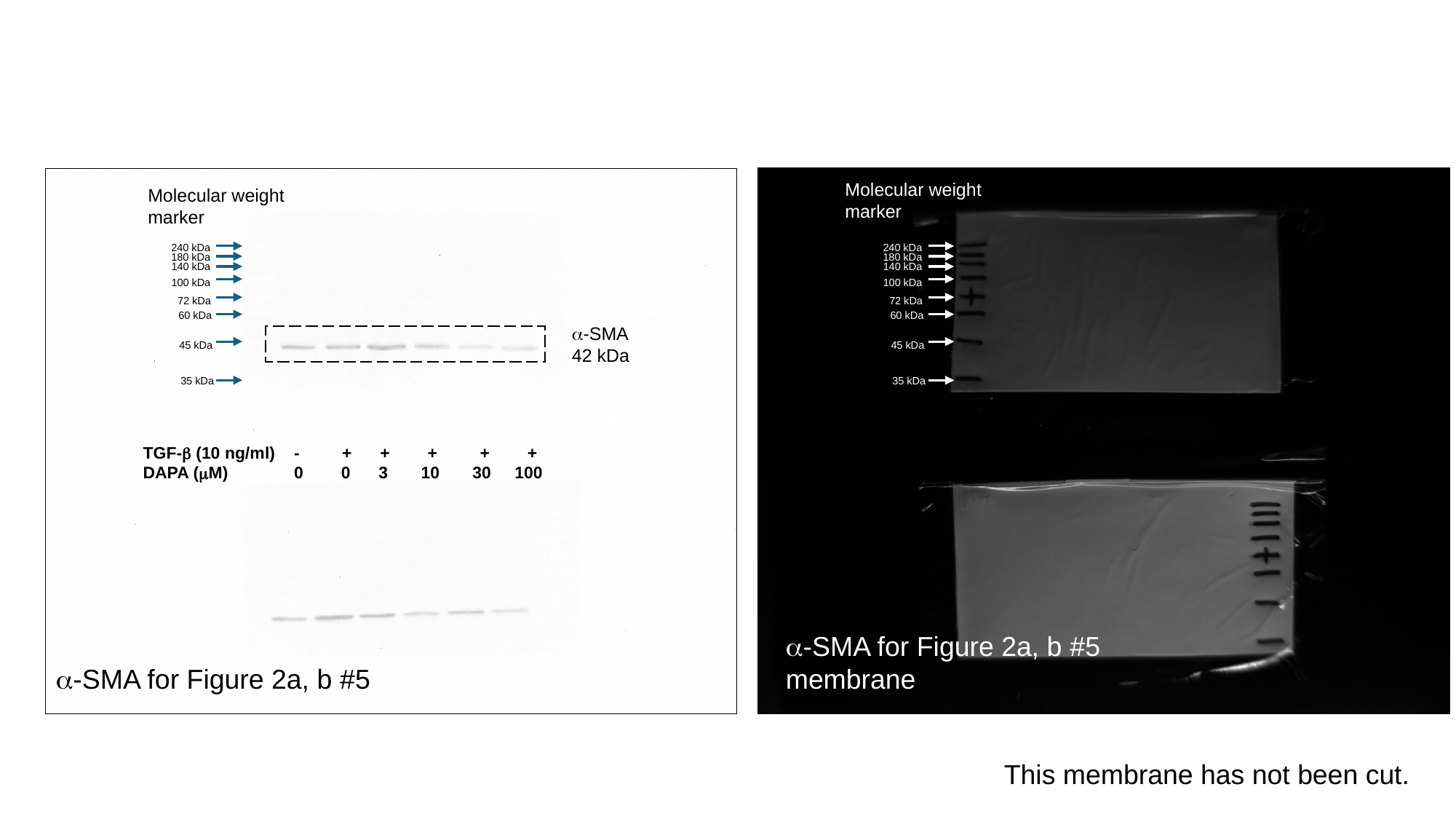

Molecular weight
marker
Molecular weight
marker
240 kDa
180 kDa
140 kDa
100 kDa
72 kDa
60 kDa
45 kDa
35 kDa
240 kDa
180 kDa
140 kDa
100 kDa
72 kDa
60 kDa
45 kDa
35 kDa
a-SMA
42 kDa
TGF-b (10 ng/ml) - + + + + +
DAPA (mM) 0 0 3 10 30 100
a-SMA for Figure 2a, b #5 membrane
a-SMA for Figure 2a, b #5
This membrane has not been cut.

## Slide 8
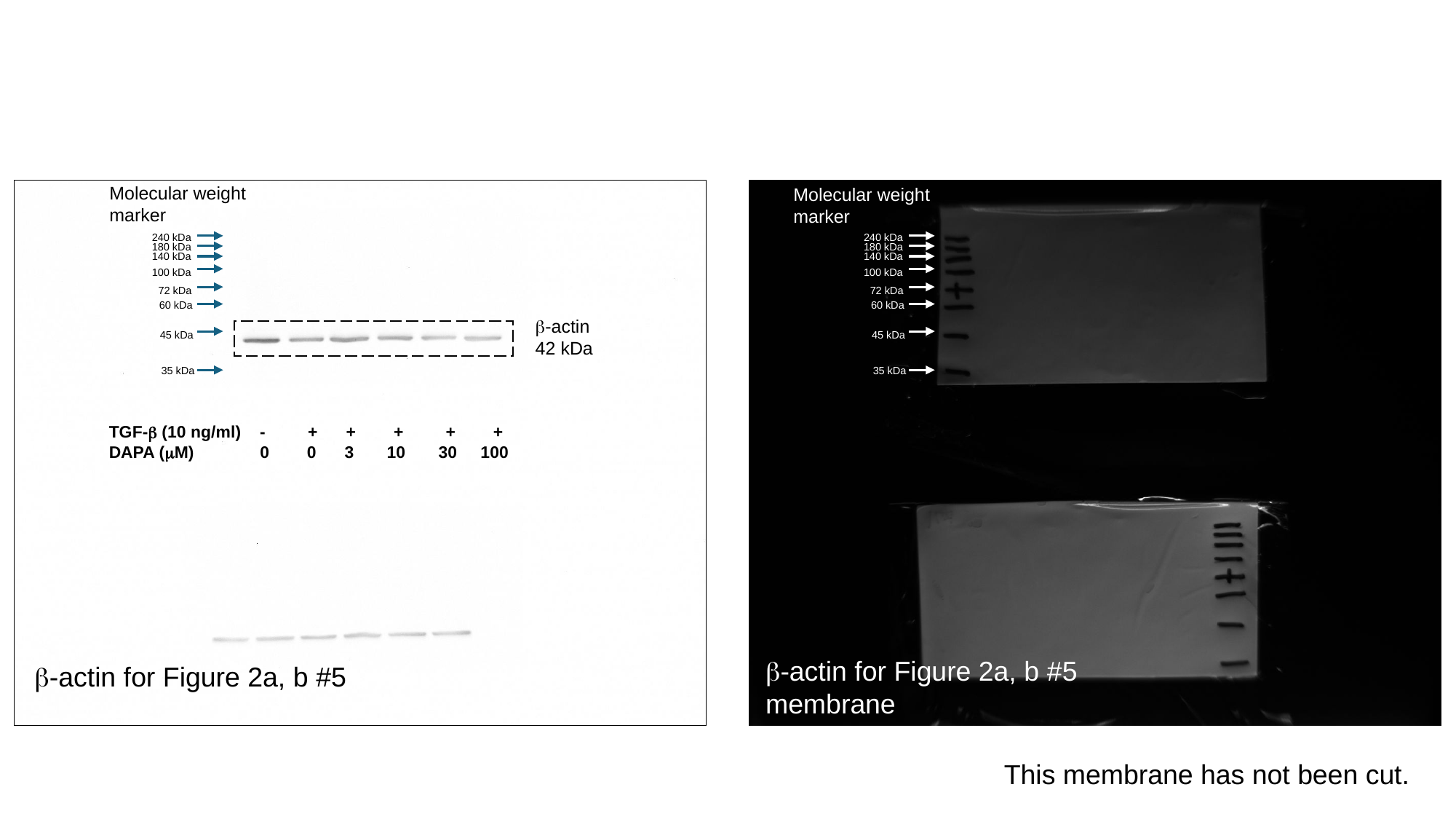

Molecular weight
marker
Molecular weight
marker
240 kDa
180 kDa
140 kDa
100 kDa
72 kDa
60 kDa
45 kDa
35 kDa
240 kDa
180 kDa
140 kDa
100 kDa
72 kDa
60 kDa
45 kDa
35 kDa
b-actin
42 kDa
TGF-b (10 ng/ml) - + + + + +
DAPA (mM) 0 0 3 10 30 100
b-actin for Figure 2a, b #5 membrane
b-actin for Figure 2a, b #5
This membrane has not been cut.

## Slide 9
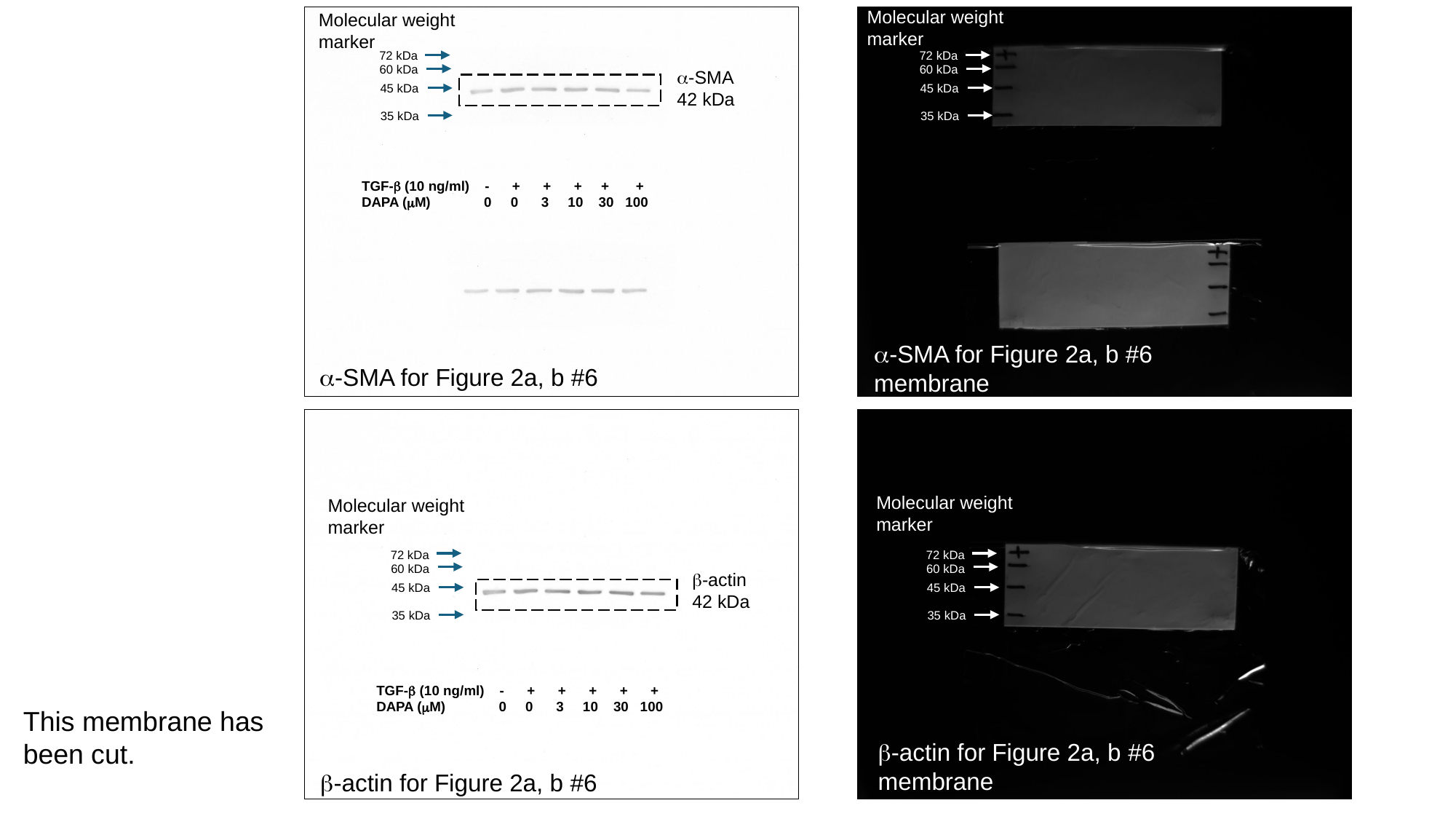

Molecular weight
marker
Molecular weight
marker
72 kDa
60 kDa
45 kDa
35 kDa
72 kDa
60 kDa
45 kDa
35 kDa
a-SMA
42 kDa
TGF-b (10 ng/ml) - + + + + +
DAPA (mM) 0 0 3 10 30 100
a-SMA for Figure 2a, b #6 membrane
a-SMA for Figure 2a, b #6
Molecular weight
marker
Molecular weight
marker
72 kDa
60 kDa
45 kDa
35 kDa
72 kDa
60 kDa
45 kDa
35 kDa
b-actin
42 kDa
TGF-b (10 ng/ml) - + + + + +
DAPA (mM) 0 0 3 10 30 100
This membrane has been cut.
b-actin for Figure 2a, b #6 membrane
b-actin for Figure 2a, b #6

## Slide 10
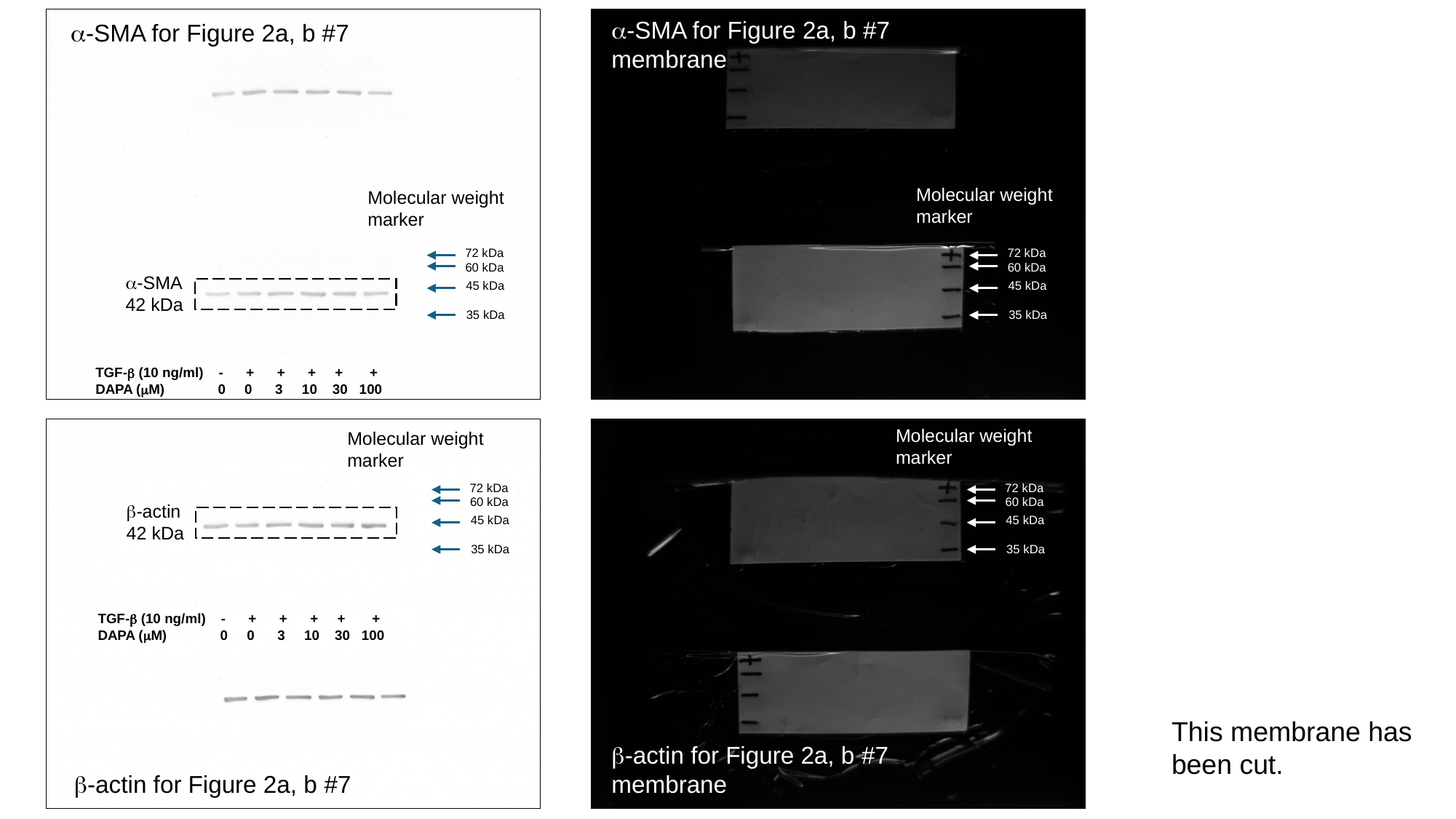

a-SMA for Figure 2a, b #7 membrane
a-SMA for Figure 2a, b #7
Molecular weight
marker
Molecular weight
marker
72 kDa
60 kDa
45 kDa
35 kDa
72 kDa
60 kDa
45 kDa
35 kDa
a-SMA
42 kDa
TGF-b (10 ng/ml) - + + + + +
DAPA (mM) 0 0 3 10 30 100
Molecular weight
marker
Molecular weight
marker
72 kDa
60 kDa
45 kDa
35 kDa
72 kDa
60 kDa
45 kDa
35 kDa
b-actin
42 kDa
TGF-b (10 ng/ml) - + + + + +
DAPA (mM) 0 0 3 10 30 100
This membrane has been cut.
b-actin for Figure 2a, b #7 membrane
b-actin for Figure 2a, b #7
