# Supplementary material for: Dapagliflozin inhibits TGF-β-induced transdifferentiation of valvular interstitial cells and mitral valvular degeneration
Source: J Mol Med (Berl). 2025 Dec 15;104(1):4. doi: 10.1007/s00109-025-02615-z (PMC12702807; doi:10.1007/s00109-025-02615-z)
Supplement: Supplementary file 5 — (PPTX 80.6 MB) [file 109_2025_2615_MOESM5_ESM.pptx]

## Slide 1
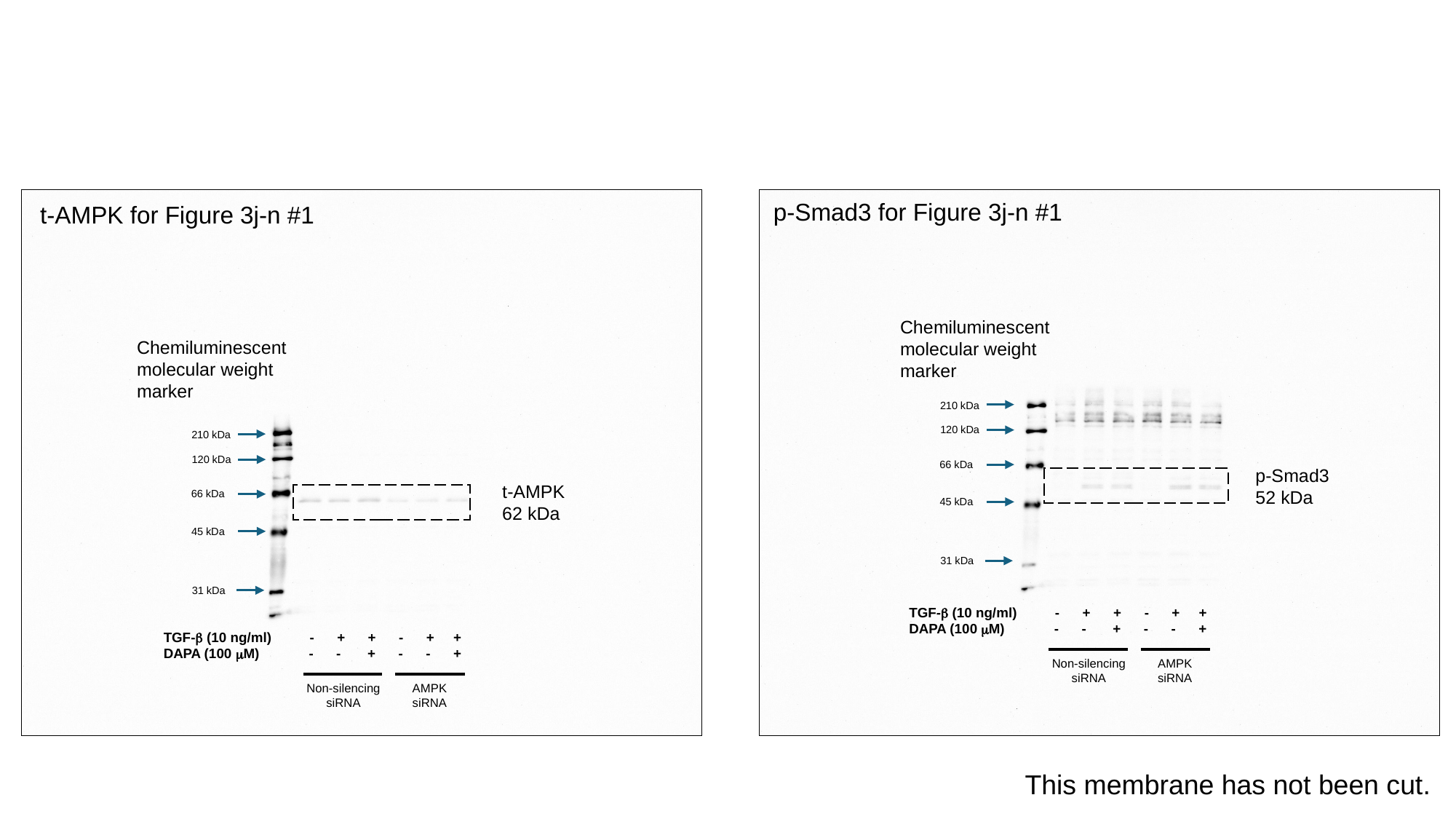

p-Smad3 for Figure 3j-n #1
t-AMPK for Figure 3j-n #1
Chemiluminescent
molecular weight
marker
Chemiluminescent
molecular weight
marker
210 kDa
120 kDa
66 kDa
45 kDa
31 kDa
210 kDa
120 kDa
66 kDa
45 kDa
31 kDa
p-Smad3
52 kDa
t-AMPK
62 kDa
TGF-b (10 ng/ml) - + + - + +
DAPA (100 mM) - - + - - +
Non-silencing
siRNA
AMPK
siRNA
TGF-b (10 ng/ml) - + + - + +
DAPA (100 mM) - - + - - +
Non-silencing
siRNA
AMPK
siRNA
This membrane has not been cut.

## Slide 2
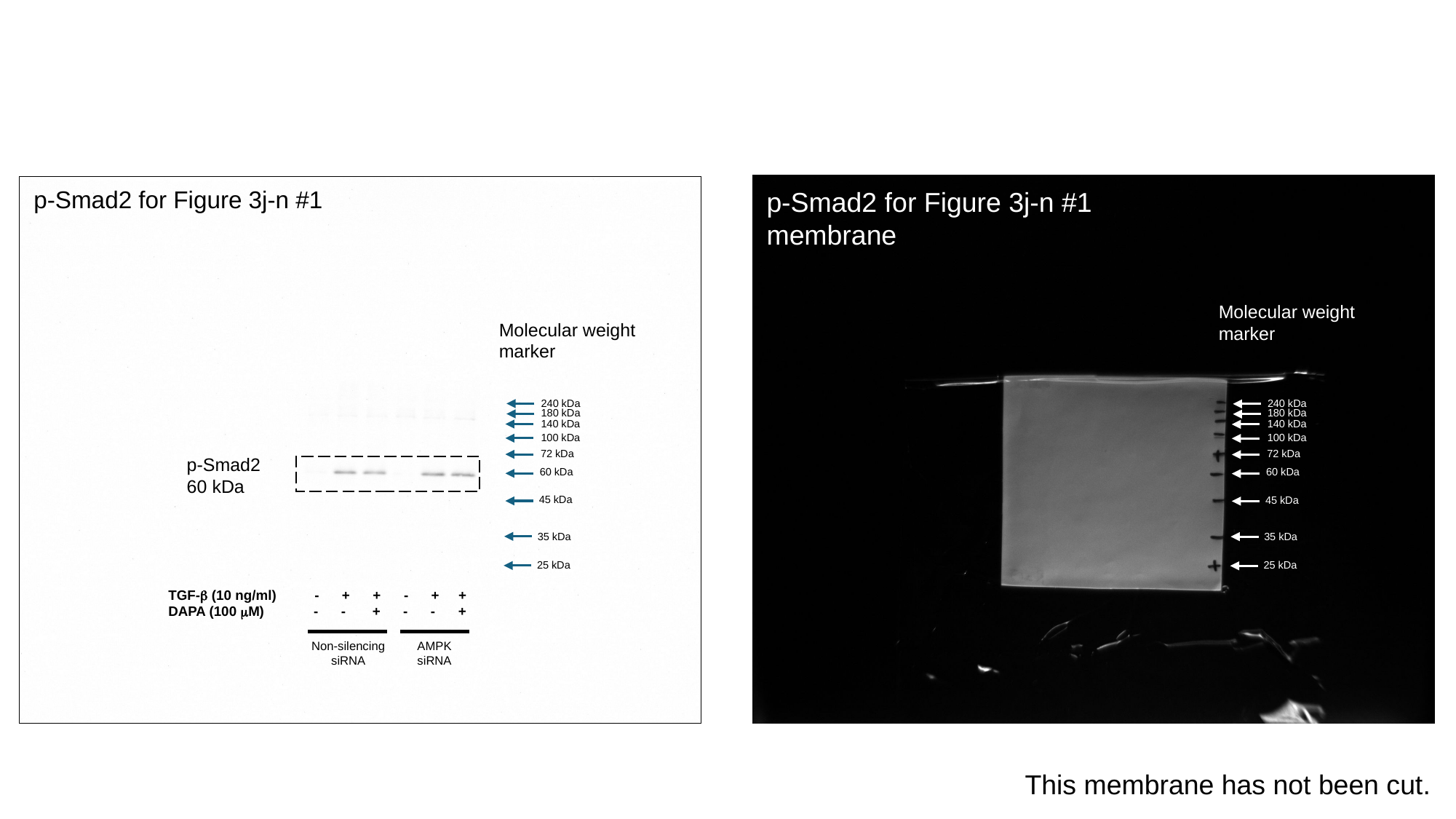

p-Smad2 for Figure 3j-n #1
p-Smad2 for Figure 3j-n #1 membrane
Molecular weight
marker
Molecular weight
marker
240 kDa
180 kDa
140 kDa
100 kDa
72 kDa
60 kDa
45 kDa
35 kDa
25 kDa
240 kDa
180 kDa
140 kDa
100 kDa
72 kDa
60 kDa
45 kDa
35 kDa
25 kDa
p-Smad2
60 kDa
TGF-b (10 ng/ml) - + + - + +
DAPA (100 mM) - - + - - +
Non-silencing
siRNA
AMPK
siRNA
This membrane has not been cut.

## Slide 3
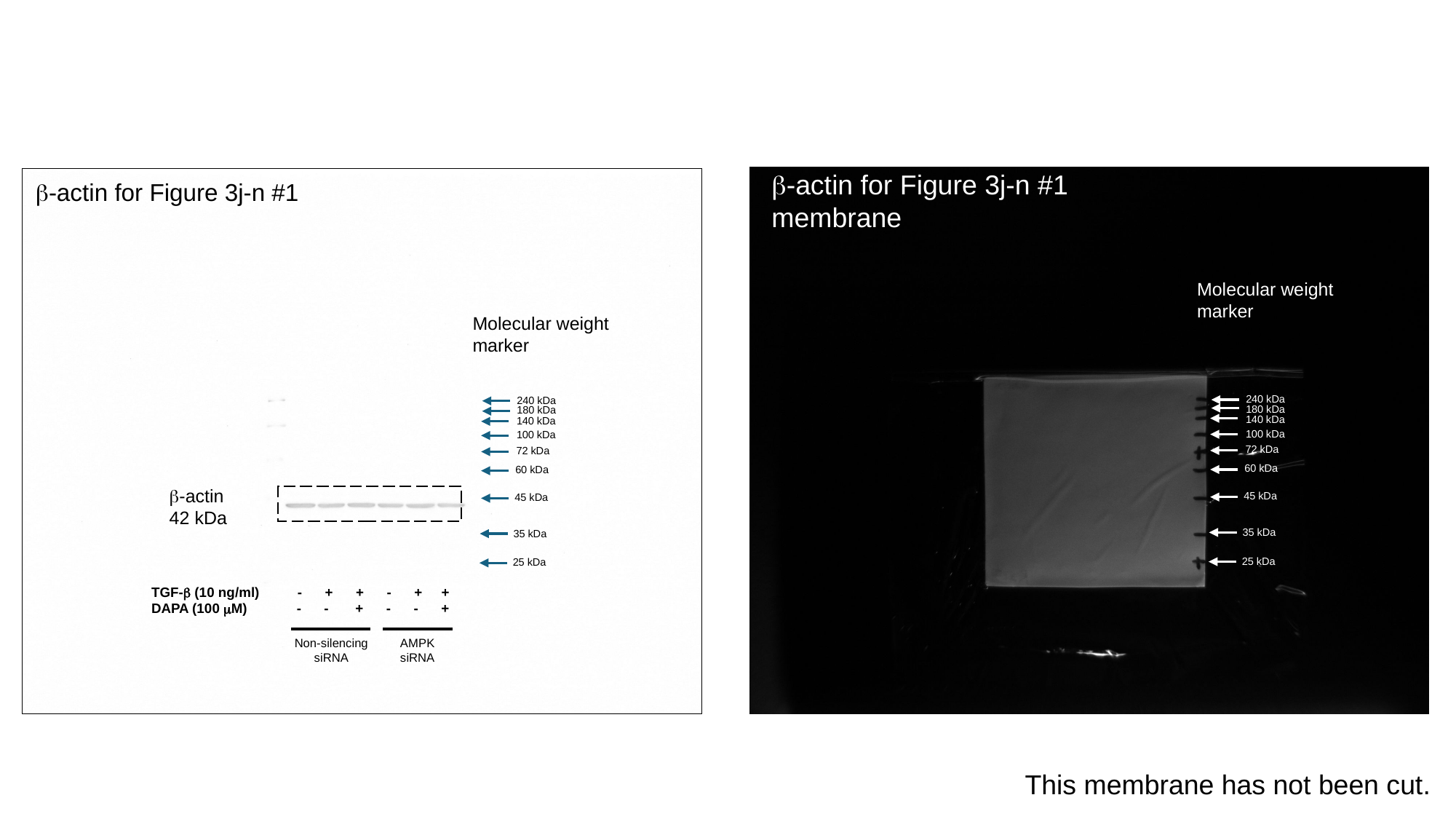

b-actin for Figure 3j-n #1 membrane
b-actin for Figure 3j-n #1
Molecular weight
marker
Molecular weight
marker
240 kDa
180 kDa
140 kDa
100 kDa
72 kDa
60 kDa
45 kDa
35 kDa
25 kDa
240 kDa
180 kDa
140 kDa
100 kDa
72 kDa
60 kDa
45 kDa
35 kDa
25 kDa
b-actin
42 kDa
TGF-b (10 ng/ml) - + + - + +
DAPA (100 mM) - - + - - +
Non-silencing
siRNA
AMPK
siRNA
This membrane has not been cut.

## Slide 4
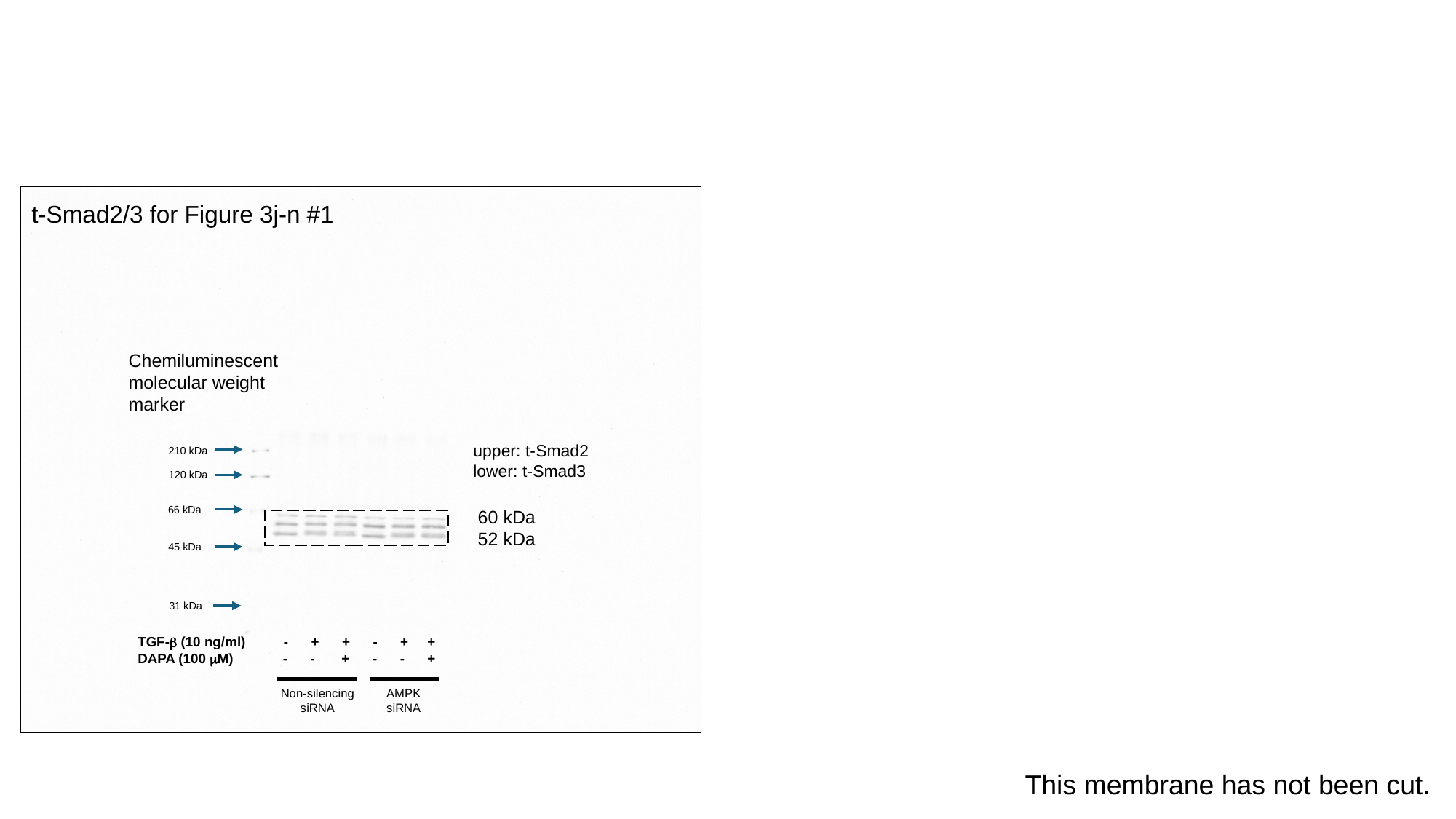

t-Smad2/3 for Figure 3j-n #1
Chemiluminescent
molecular weight
marker
upper: t-Smad2
lower: t-Smad3
210 kDa
120 kDa
66 kDa
45 kDa
31 kDa
60 kDa
52 kDa
TGF-b (10 ng/ml) - + + - + +
DAPA (100 mM) - - + - - +
Non-silencing
siRNA
AMPK
siRNA
This membrane has not been cut.

## Slide 5
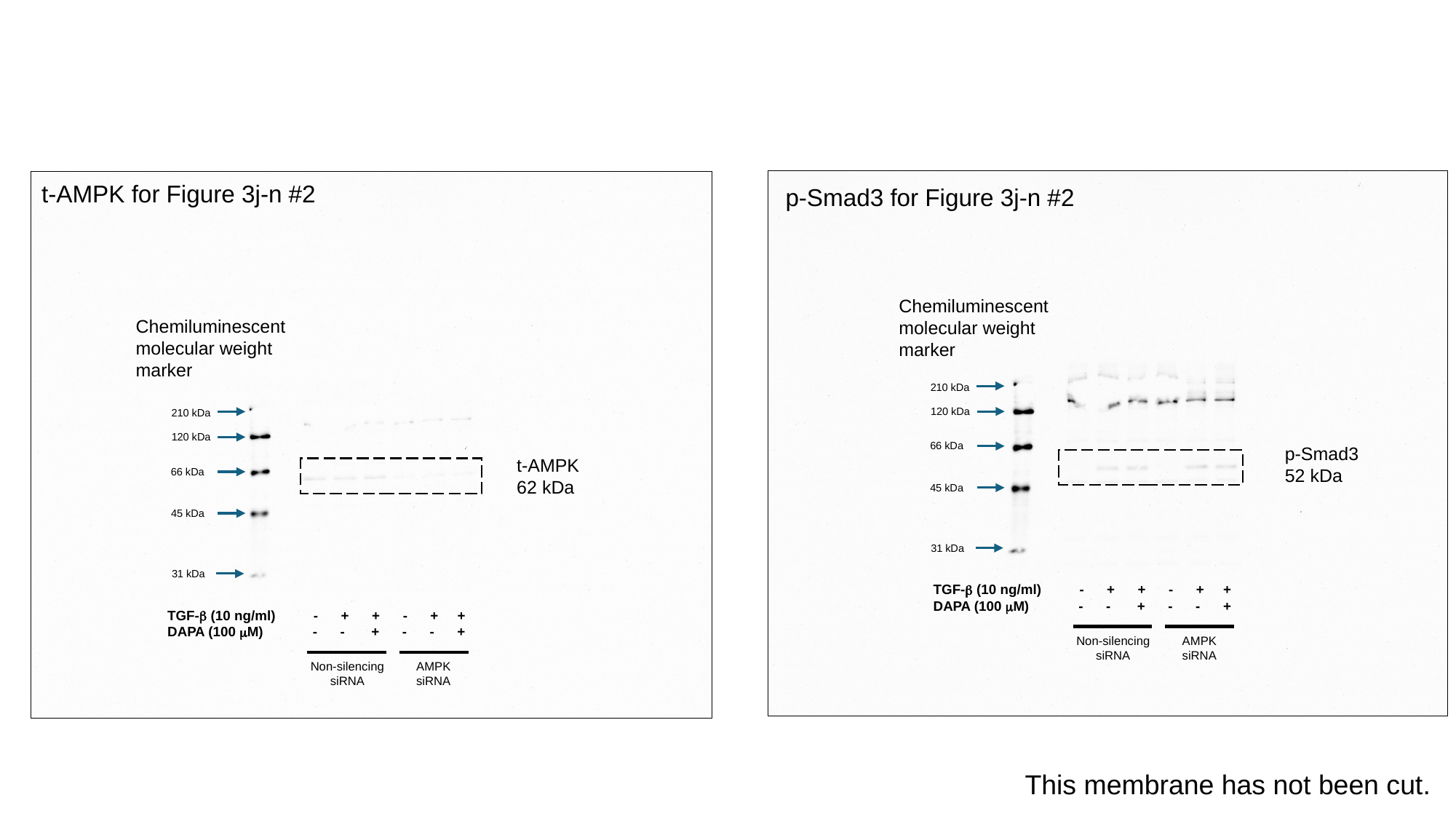

t-AMPK for Figure 3j-n #2
p-Smad3 for Figure 3j-n #2
Chemiluminescent
molecular weight
marker
Chemiluminescent
molecular weight
marker
210 kDa
120 kDa
66 kDa
45 kDa
31 kDa
210 kDa
120 kDa
66 kDa
45 kDa
31 kDa
p-Smad3
52 kDa
t-AMPK
62 kDa
TGF-b (10 ng/ml) - + + - + +
DAPA (100 mM) - - + - - +
Non-silencing
siRNA
AMPK
siRNA
TGF-b (10 ng/ml) - + + - + +
DAPA (100 mM) - - + - - +
Non-silencing
siRNA
AMPK
siRNA
This membrane has not been cut.

## Slide 6
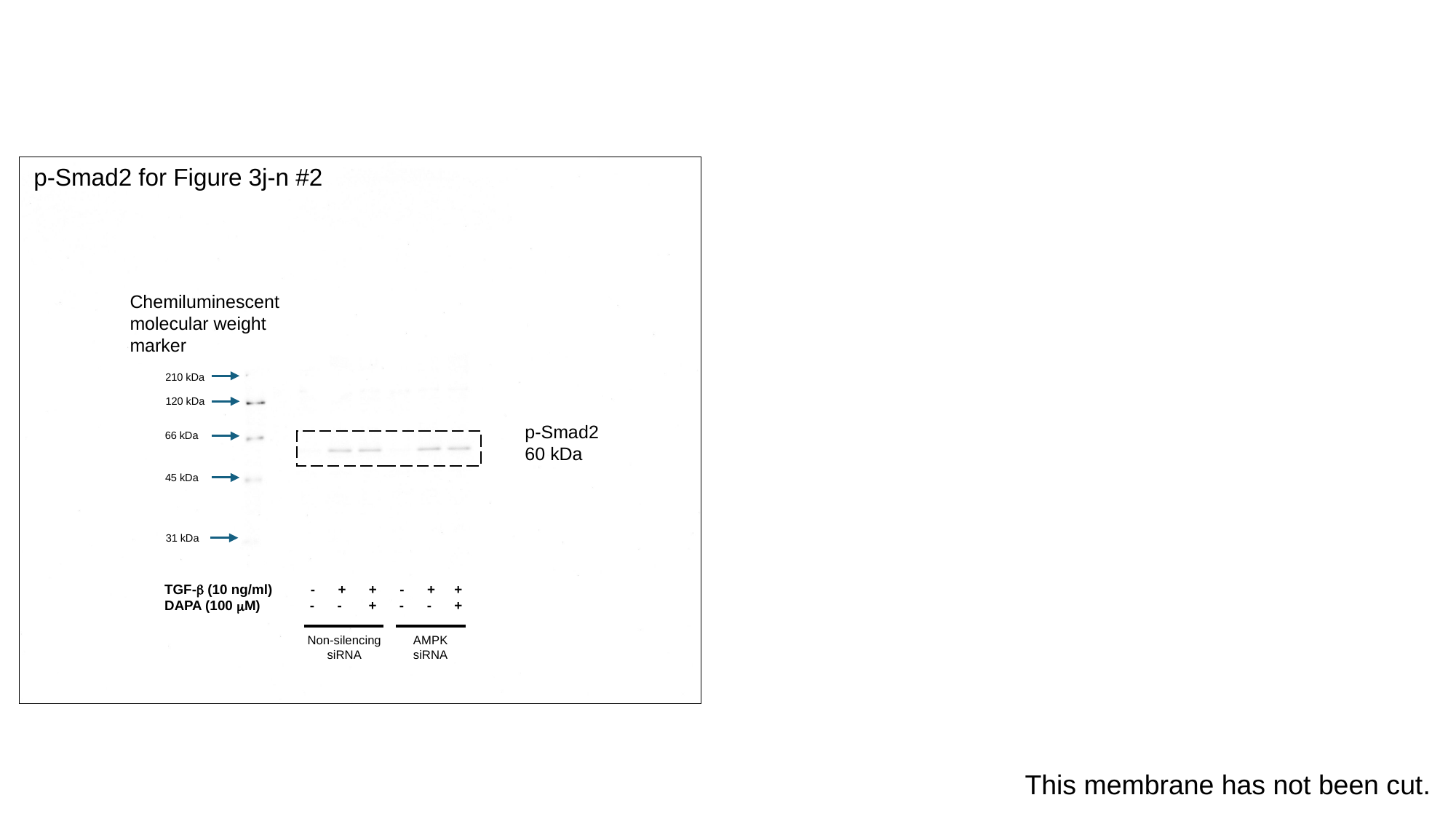

p-Smad2 for Figure 3j-n #2
Chemiluminescent
molecular weight
marker
210 kDa
120 kDa
66 kDa
45 kDa
31 kDa
p-Smad2
60 kDa
TGF-b (10 ng/ml) - + + - + +
DAPA (100 mM) - - + - - +
Non-silencing
siRNA
AMPK
siRNA
This membrane has not been cut.

## Slide 7
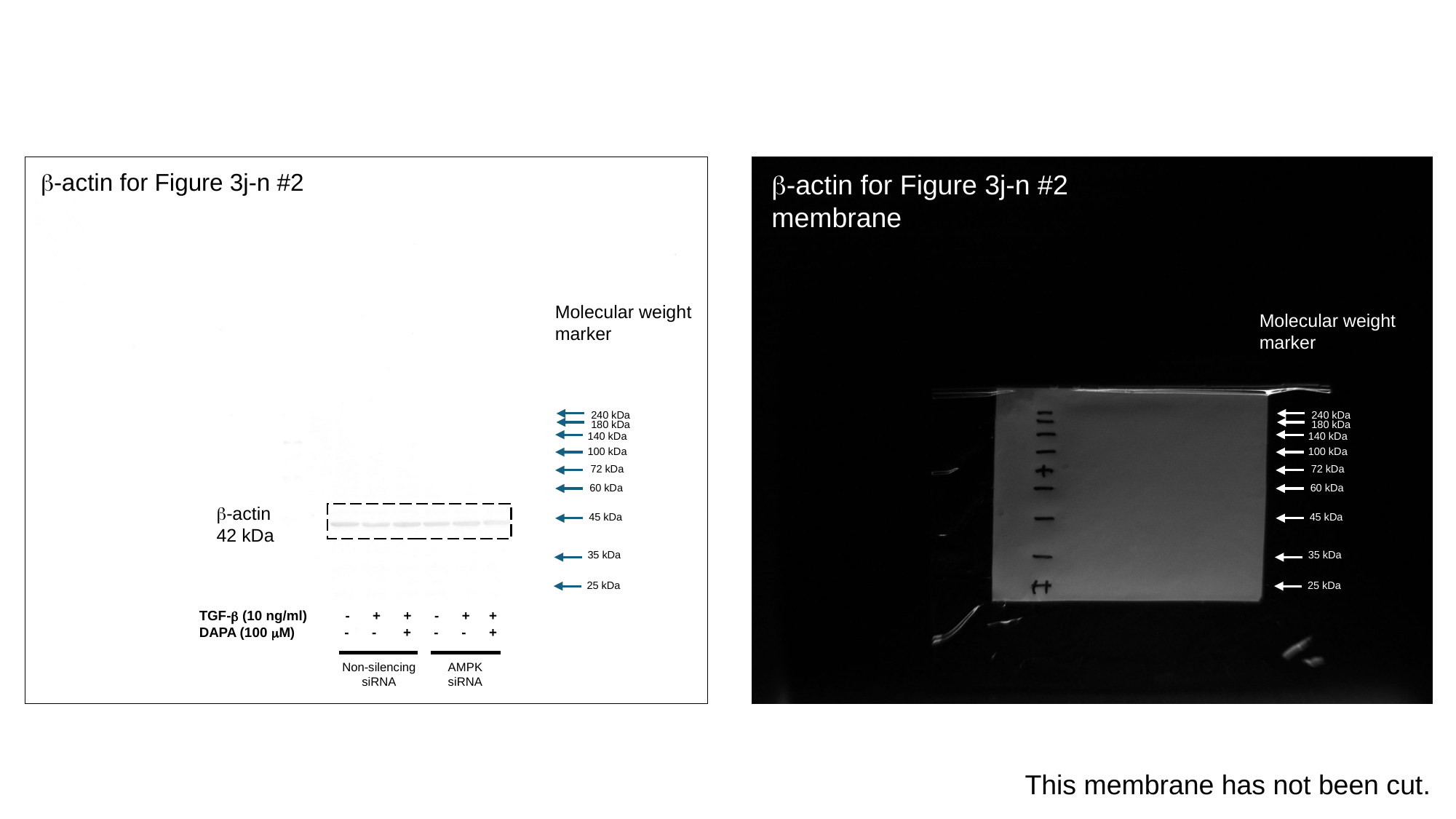

b-actin for Figure 3j-n #2
b-actin for Figure 3j-n #2 membrane
Molecular weight
marker
Molecular weight
marker
240 kDa
180 kDa
140 kDa
100 kDa
72 kDa
60 kDa
45 kDa
35 kDa
25 kDa
240 kDa
180 kDa
140 kDa
100 kDa
72 kDa
60 kDa
45 kDa
35 kDa
25 kDa
b-actin
42 kDa
TGF-b (10 ng/ml) - + + - + +
DAPA (100 mM) - - + - - +
Non-silencing
siRNA
AMPK
siRNA
This membrane has not been cut.

## Slide 8
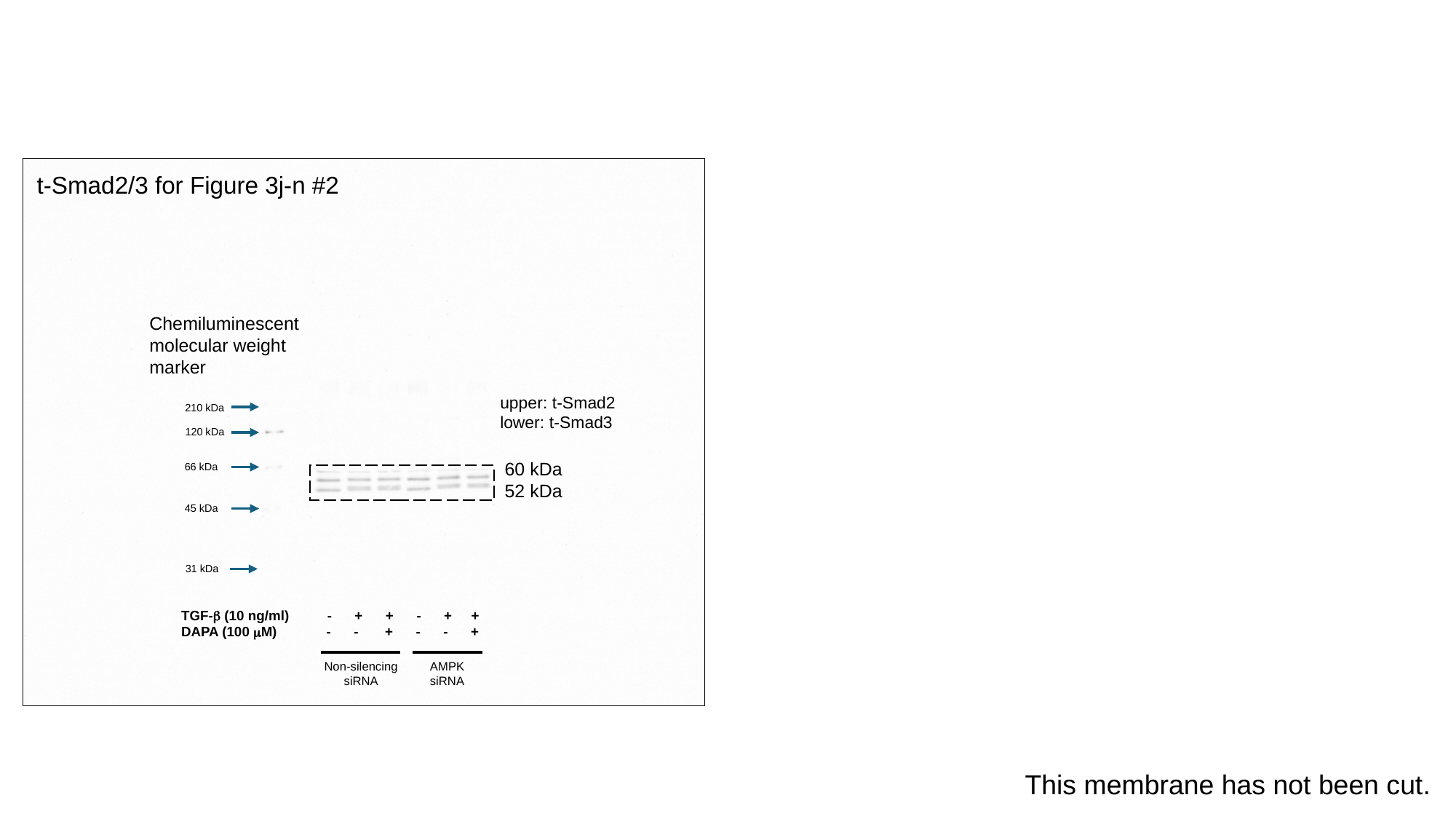

t-Smad2/3 for Figure 3j-n #2
Chemiluminescent
molecular weight
marker
upper: t-Smad2
lower: t-Smad3
210 kDa
120 kDa
66 kDa
45 kDa
31 kDa
60 kDa
52 kDa
TGF-b (10 ng/ml) - + + - + +
DAPA (100 mM) - - + - - +
Non-silencing
siRNA
AMPK
siRNA
This membrane has not been cut.

## Slide 9
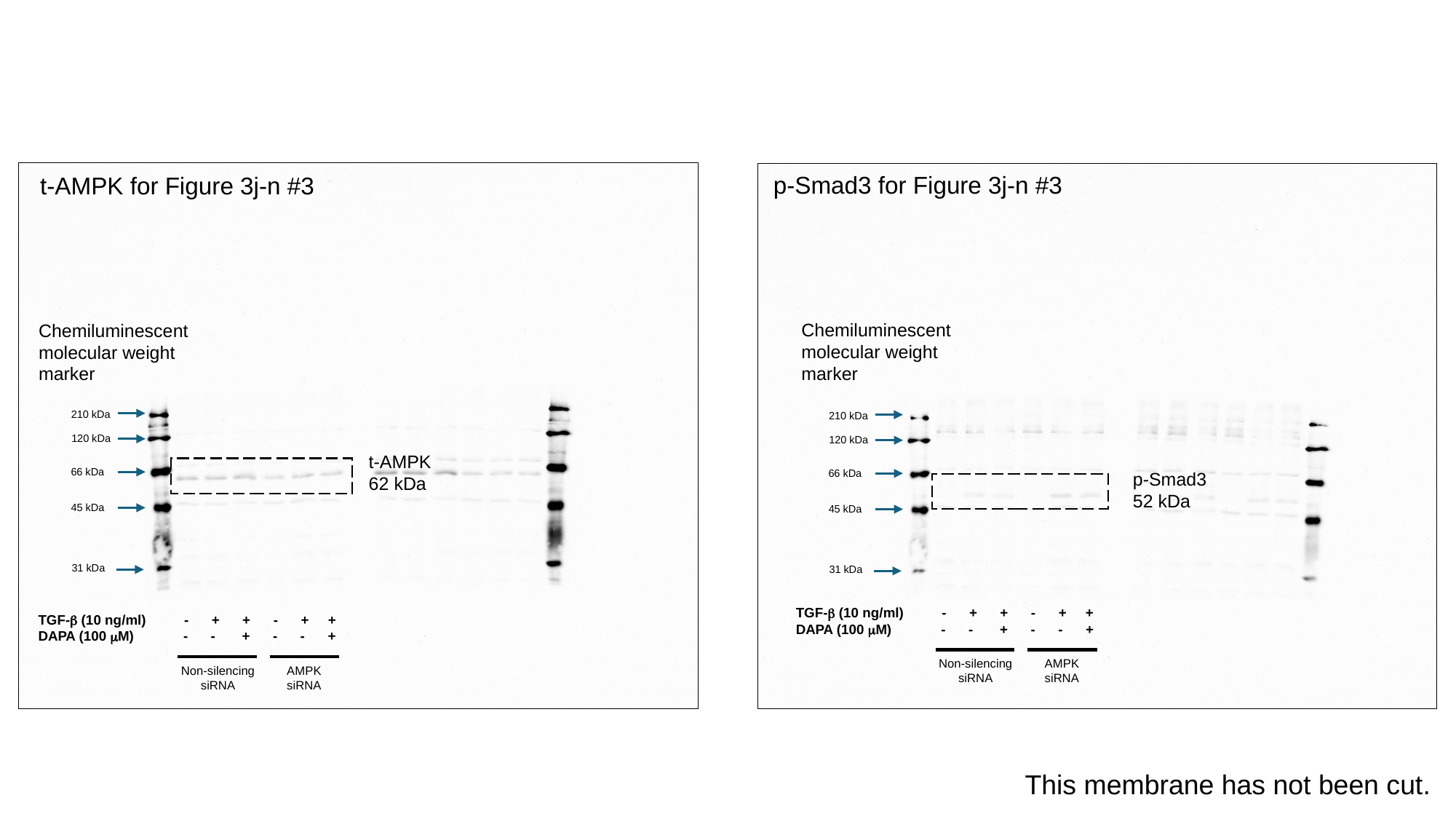

p-Smad3 for Figure 3j-n #3
t-AMPK for Figure 3j-n #3
Chemiluminescent
molecular weight
marker
Chemiluminescent
molecular weight
marker
210 kDa
120 kDa
66 kDa
45 kDa
31 kDa
210 kDa
120 kDa
66 kDa
45 kDa
31 kDa
t-AMPK
62 kDa
p-Smad3
52 kDa
TGF-b (10 ng/ml) - + + - + +
DAPA (100 mM) - - + - - +
Non-silencing
siRNA
AMPK
siRNA
TGF-b (10 ng/ml) - + + - + +
DAPA (100 mM) - - + - - +
Non-silencing
siRNA
AMPK
siRNA
This membrane has not been cut.

## Slide 10
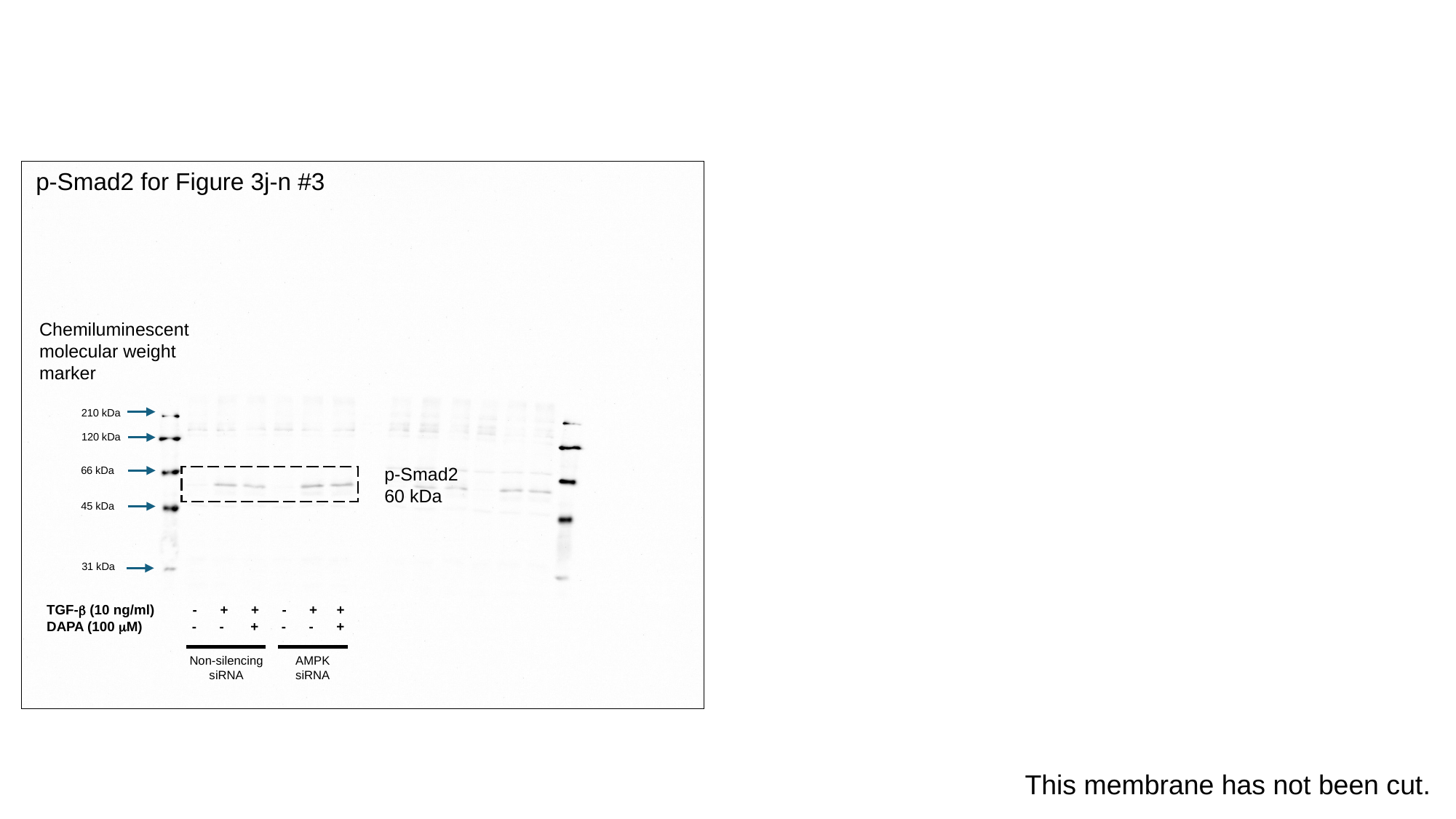

p-Smad2 for Figure 3j-n #3
Chemiluminescent
molecular weight
marker
210 kDa
120 kDa
66 kDa
45 kDa
31 kDa
p-Smad2
60 kDa
TGF-b (10 ng/ml) - + + - + +
DAPA (100 mM) - - + - - +
Non-silencing
siRNA
AMPK
siRNA
This membrane has not been cut.

## Slide 11
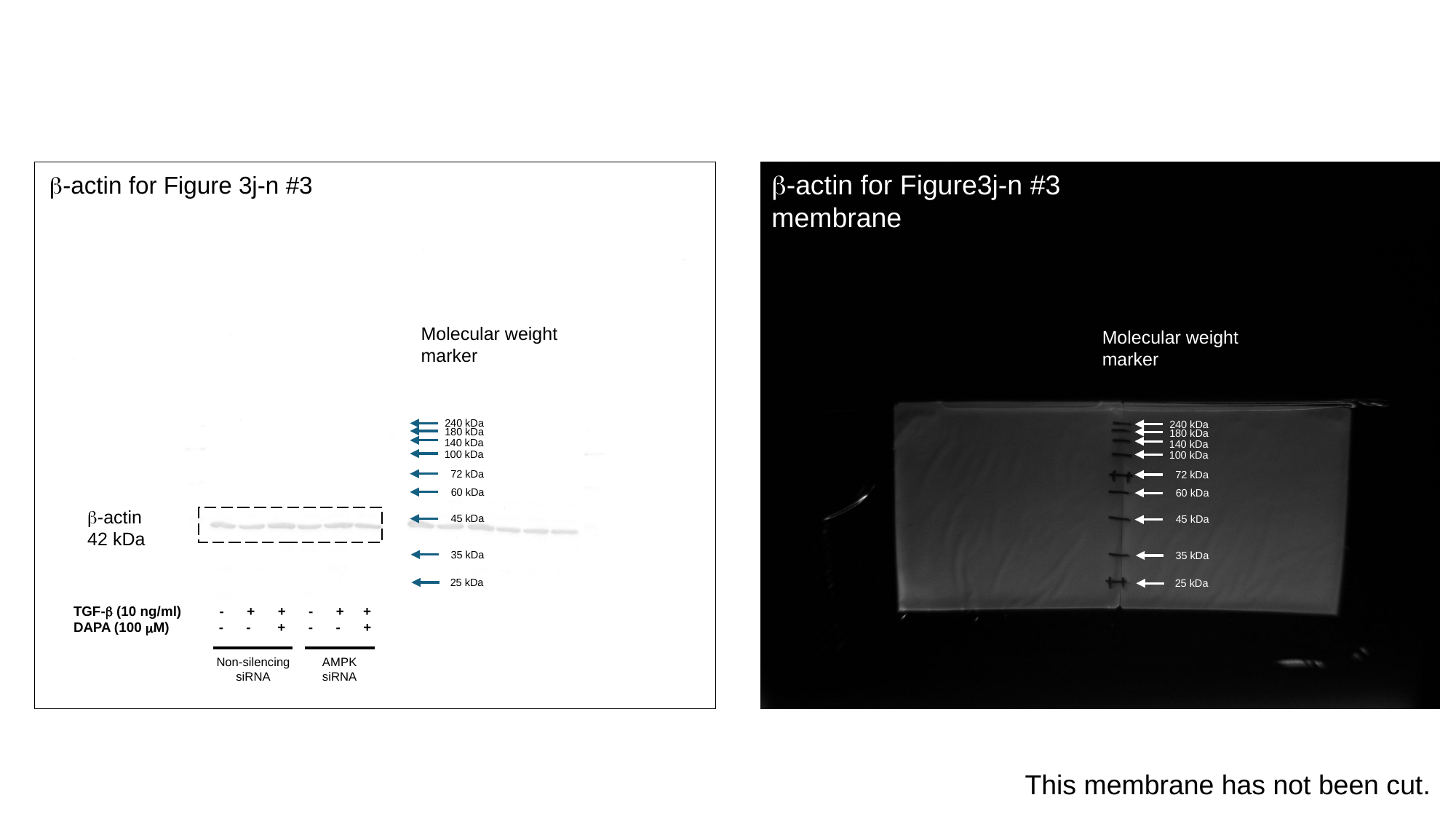

b-actin for Figure3j-n #3 membrane
b-actin for Figure 3j-n #3
Molecular weight
marker
Molecular weight
marker
240 kDa
180 kDa
140 kDa
100 kDa
72 kDa
60 kDa
45 kDa
35 kDa
25 kDa
240 kDa
180 kDa
140 kDa
100 kDa
72 kDa
60 kDa
45 kDa
35 kDa
25 kDa
b-actin
42 kDa
TGF-b (10 ng/ml) - + + - + +
DAPA (100 mM) - - + - - +
Non-silencing
siRNA
AMPK
siRNA
This membrane has not been cut.

## Slide 12
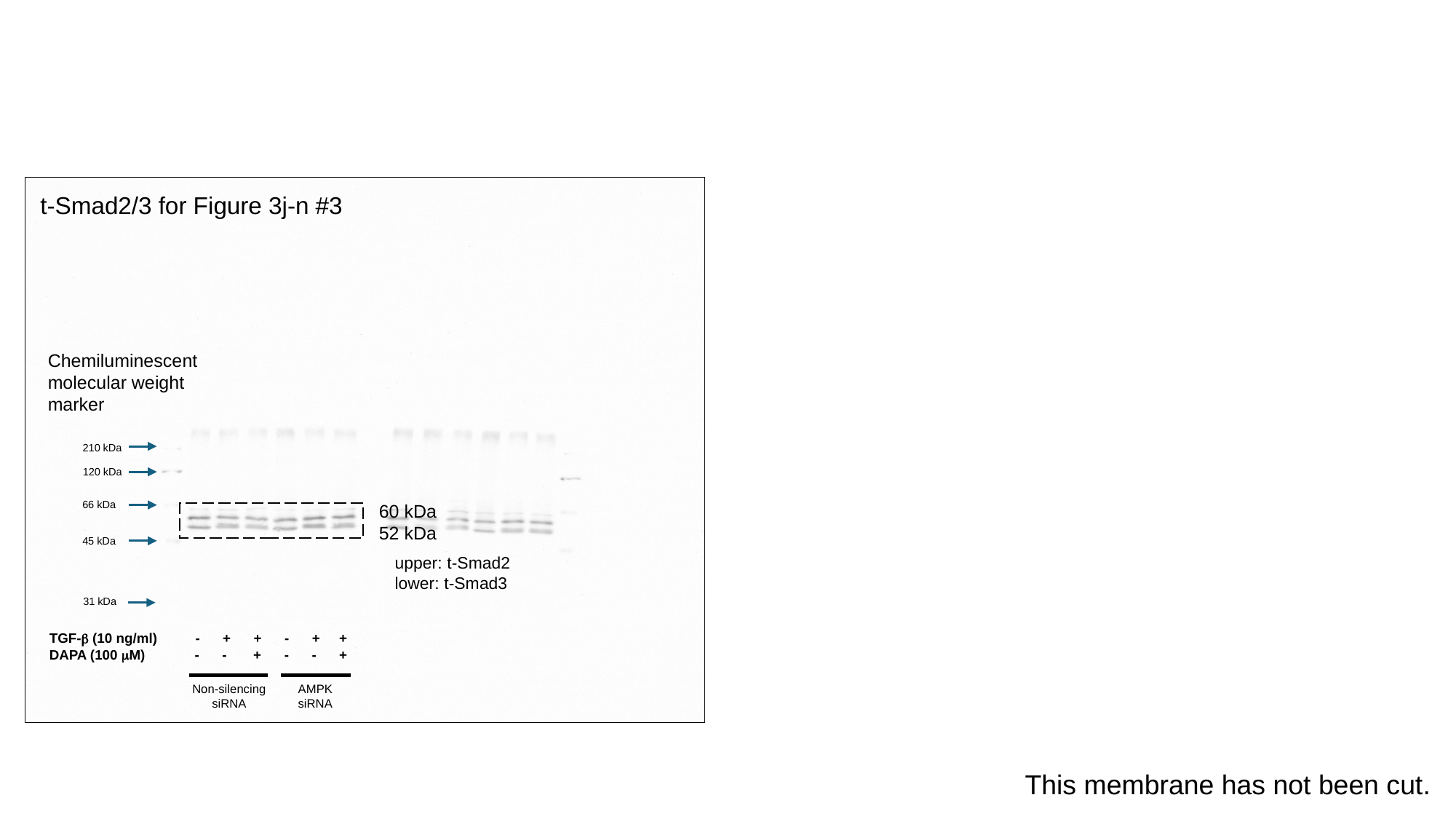

t-Smad2/3 for Figure 3j-n #3
Chemiluminescent
molecular weight
marker
210 kDa
120 kDa
66 kDa
45 kDa
31 kDa
60 kDa
52 kDa
upper: t-Smad2
lower: t-Smad3
TGF-b (10 ng/ml) - + + - + +
DAPA (100 mM) - - + - - +
Non-silencing
siRNA
AMPK
siRNA
This membrane has not been cut.

## Slide 13
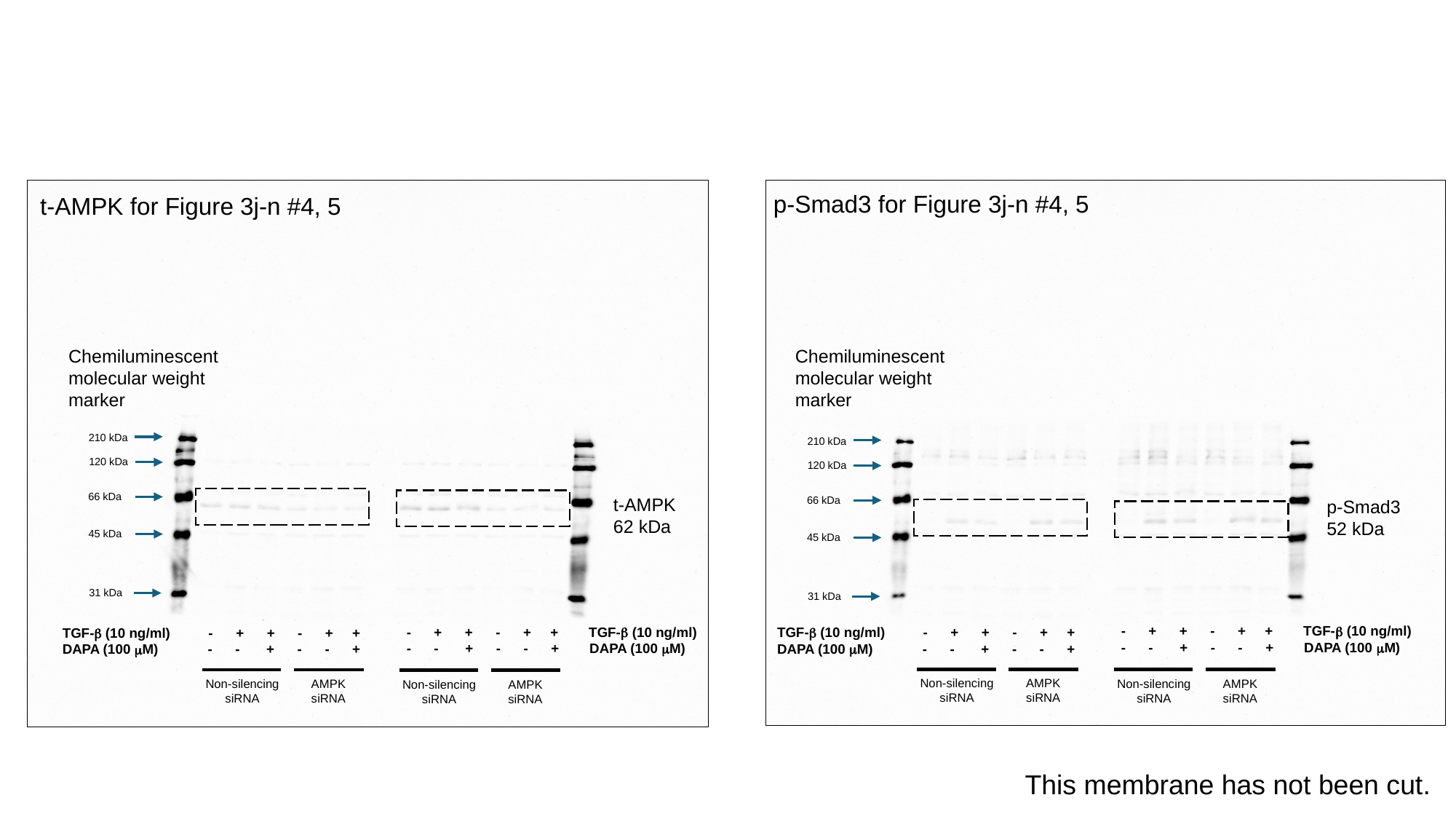

p-Smad3 for Figure 3j-n #4, 5
t-AMPK for Figure 3j-n #4, 5
Chemiluminescent
molecular weight
marker
Chemiluminescent
molecular weight
marker
210 kDa
120 kDa
66 kDa
45 kDa
31 kDa
210 kDa
120 kDa
66 kDa
45 kDa
31 kDa
t-AMPK
62 kDa
p-Smad3
52 kDa
- + + - + + TGF-b (10 ng/ml)
- - + - - + DAPA (100 mM)
Non-silencing
siRNA
AMPK
siRNA
- + + - + + TGF-b (10 ng/ml)
- - + - - + DAPA (100 mM)
Non-silencing
siRNA
AMPK
siRNA
TGF-b (10 ng/ml) - + + - + +
DAPA (100 mM) - - + - - +
Non-silencing
siRNA
AMPK
siRNA
TGF-b (10 ng/ml) - + + - + +
DAPA (100 mM) - - + - - +
Non-silencing
siRNA
AMPK
siRNA
This membrane has not been cut.

## Slide 14
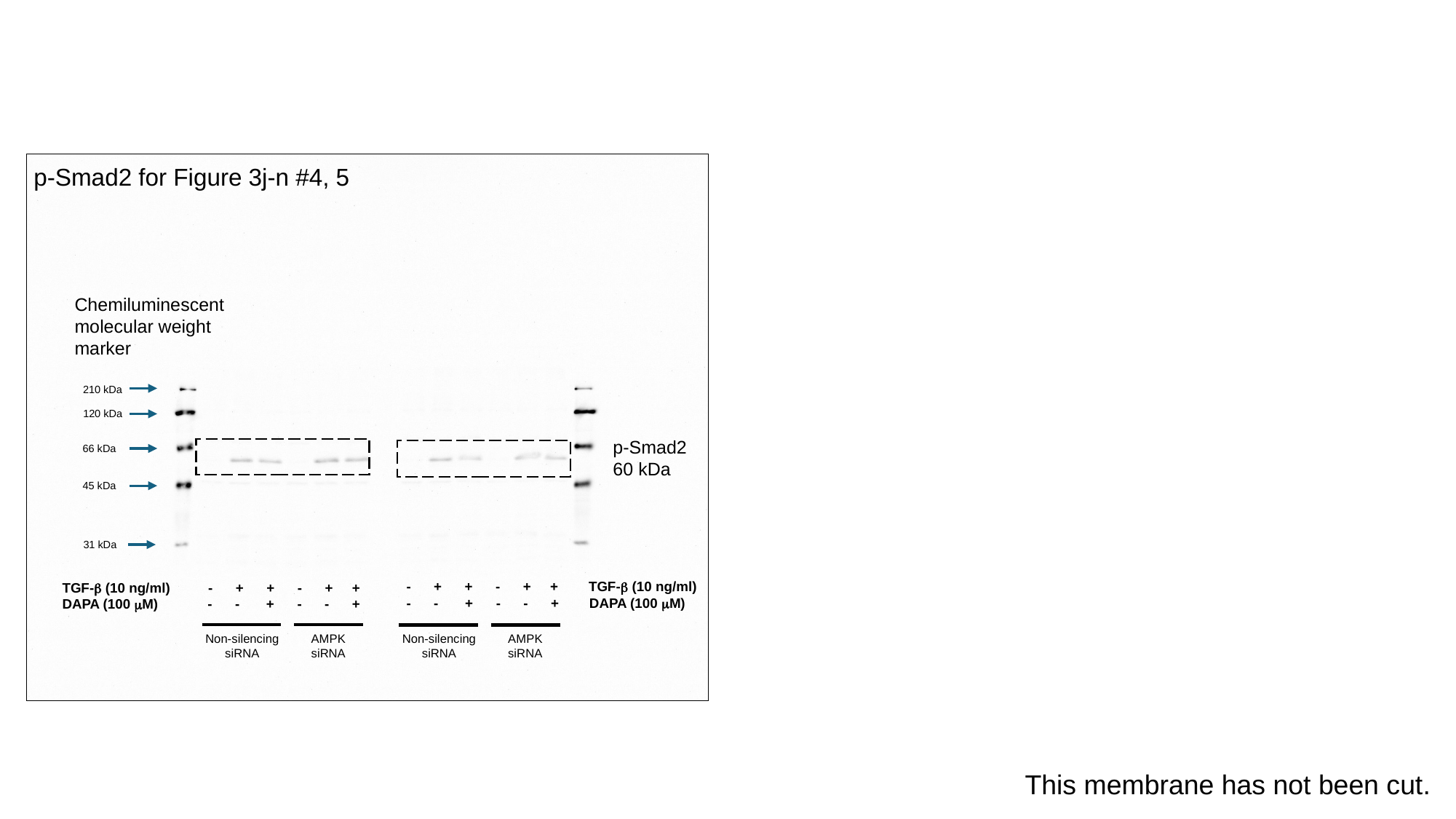

p-Smad2 for Figure 3j-n #4, 5
Chemiluminescent
molecular weight
marker
210 kDa
120 kDa
66 kDa
45 kDa
31 kDa
p-Smad2
60 kDa
- + + - + + TGF-b (10 ng/ml)
- - + - - + DAPA (100 mM)
Non-silencing
siRNA
AMPK
siRNA
TGF-b (10 ng/ml) - + + - + +
DAPA (100 mM) - - + - - +
Non-silencing
siRNA
AMPK
siRNA
This membrane has not been cut.

## Slide 15
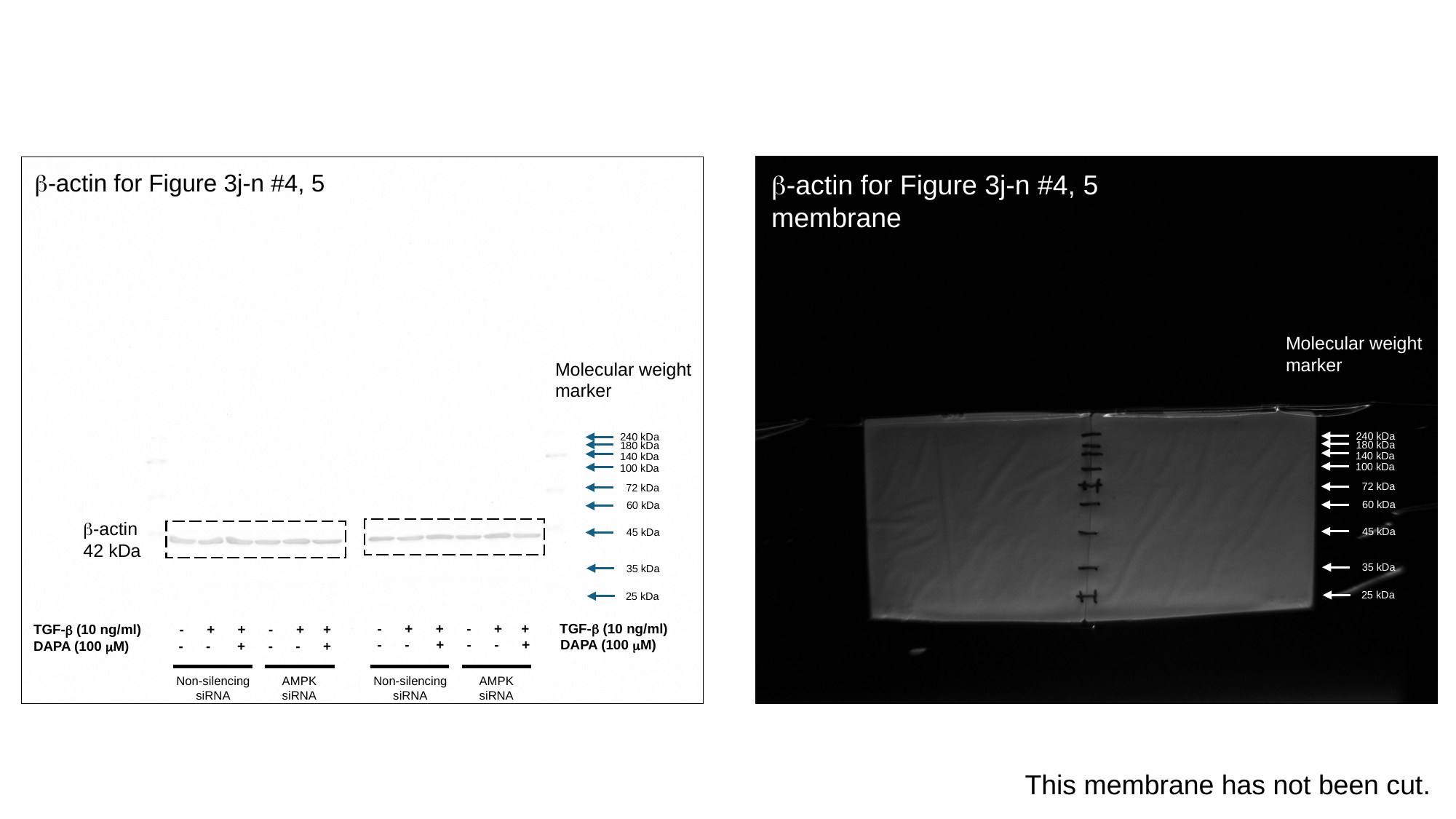

b-actin for Figure 3j-n #4, 5 membrane
b-actin for Figure 3j-n #4, 5
Molecular weight
marker
Molecular weight
marker
240 kDa
180 kDa
140 kDa
100 kDa
72 kDa
60 kDa
45 kDa
35 kDa
25 kDa
240 kDa
180 kDa
140 kDa
100 kDa
72 kDa
60 kDa
45 kDa
35 kDa
25 kDa
b-actin
42 kDa
- + + - + + TGF-b (10 ng/ml)
- - + - - + DAPA (100 mM)
Non-silencing
siRNA
AMPK
siRNA
TGF-b (10 ng/ml) - + + - + +
DAPA (100 mM) - - + - - +
Non-silencing
siRNA
AMPK
siRNA
This membrane has not been cut.

## Slide 16
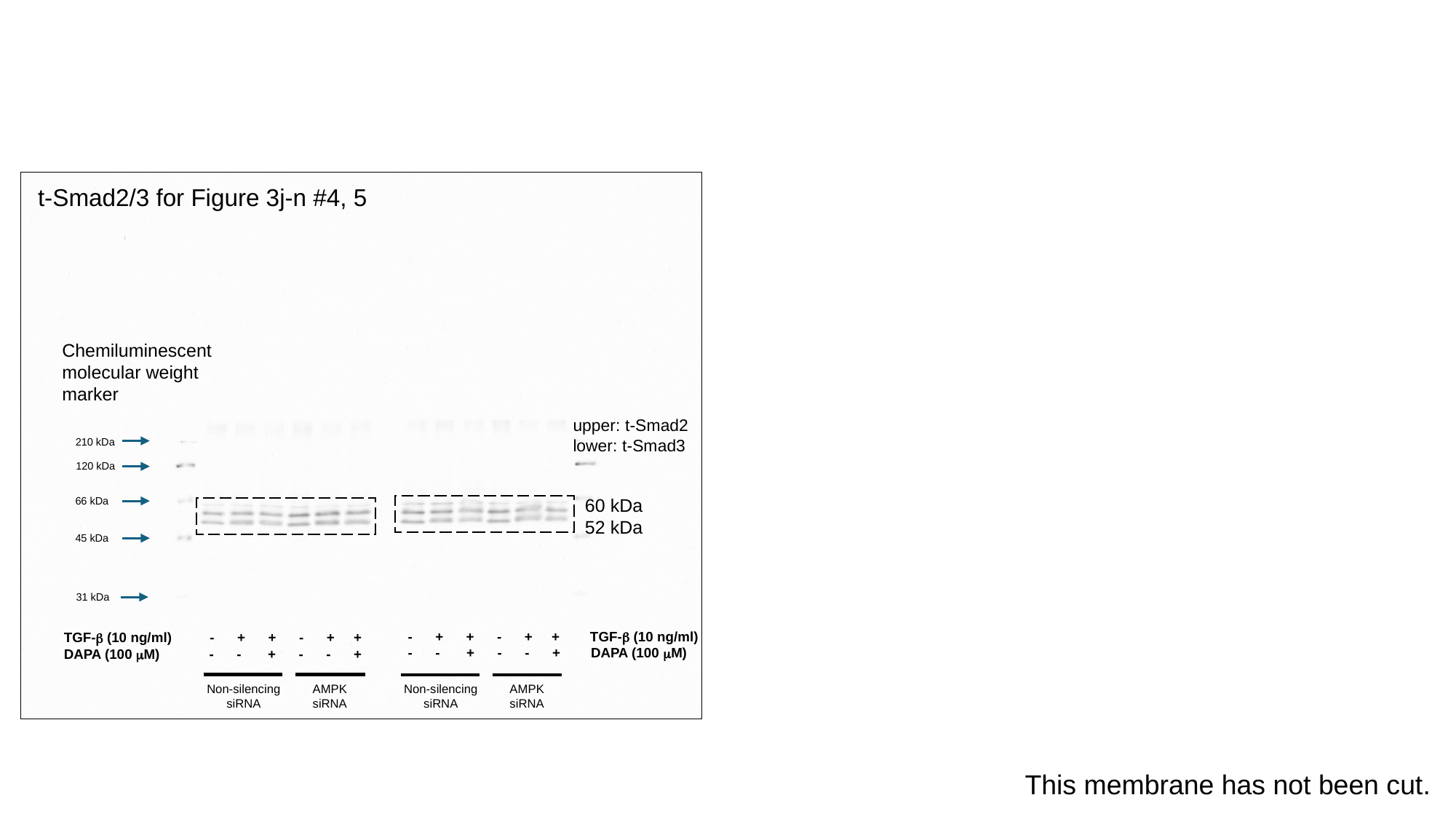

t-Smad2/3 for Figure 3j-n #4, 5
Chemiluminescent
molecular weight
marker
upper: t-Smad2
lower: t-Smad3
210 kDa
120 kDa
66 kDa
45 kDa
31 kDa
60 kDa
52 kDa
- + + - + + TGF-b (10 ng/ml)
- - + - - + DAPA (100 mM)
Non-silencing
siRNA
AMPK
siRNA
TGF-b (10 ng/ml) - + + - + +
DAPA (100 mM) - - + - - +
Non-silencing
siRNA
AMPK
siRNA
This membrane has not been cut.
